# Metabolic Modeling Combined With Machine Learning Integrates Longitudinal Data and Identifies the Origin of LXR-Induced Hepatic Steatosis

**DOI:** 10.3389/fbioe.2020.536957

**Published:** 2021-02-16

**Authors:** Natal A. W. van Riel, Christian A. Tiemann, Peter A. J. Hilbers, Albert K. Groen

**Affiliations:** ^1^Department of Biomedical Engineering, Eindhoven University of Technology, Eindhoven, Netherlands; ^2^Department of Vascular Medicine, Amsterdam UMC, Amsterdam, Netherlands; ^3^Maastricht Centre for Systems Biology, Maastricht University, Maastricht, Netherlands; ^4^Department of Laboratory Medicine, University Medical Center Groningen, Groningen, Netherlands

**Keywords:** longitudinal trajectory modeling, regularization, cholesterol, LXR agonist, systems biology, machine learning, mechanistic modeling, uncertainty quantification

## Abstract

Temporal multi-omics data can provide information about the dynamics of disease development and therapeutic response. However, statistical analysis of high-dimensional time-series data is challenging. Here we develop a novel approach to model temporal metabolomic and transcriptomic data by combining machine learning with metabolic models. ADAPT (Analysis of Dynamic Adaptations in Parameter Trajectories) performs metabolic trajectory modeling by introducing time-dependent parameters in differential equation models of metabolic systems. ADAPT translates structural uncertainty in the model, such as missing information about regulation, into a parameter estimation problem that is solved by iterative learning. We have now extended ADAPT to include both metabolic and transcriptomic time-series data by introducing a regularization function in the learning algorithm. The ADAPT learning algorithm was (re)formulated as a multi-objective optimization problem in which the estimation of trajectories of metabolic parameters is constrained by the metabolite data and refined by gene expression data. ADAPT was applied to a model of hepatic lipid and plasma lipoprotein metabolism to predict metabolic adaptations that are induced upon pharmacological treatment of mice by a Liver X receptor (LXR) agonist. We investigated the excessive accumulation of triglycerides (TG) in the liver resulting in the development of hepatic steatosis. ADAPT predicted that hepatic TG accumulation after LXR activation originates for 80% from an increased influx of free fatty acids. The model also correctly estimated that TG was stored in the cytosol rather than transferred to nascent very-low density lipoproteins. Through model-based integration of temporal metabolic and gene expression data we discovered that increased free fatty acid influx instead of *de novo* lipogenesis is the main driver of LXR-induced hepatic steatosis. This study illustrates how ADAPT provides estimates for biomedically important parameters that cannot be measured directly, explaining (side-)effects of pharmacological treatment with LXR agonists.

## 1. Introduction

Dynamic responses contain important information about the behavior of biological systems. For example, data from continuous glucose monitoring has been used to identify characteristic patterns in glucose dynamics (Hall et al., 2018). Statistical modeling of time-series data using machine learning works well if the number of samples (individuals) in the dataset is large and the number of outcome variables is (relatively) small. For example, Latent Class Trajectory Analysis has been applied for time-series modeling of glucose measurements obtained during an oral glucose tolerance test (Hulman et al., 2018), thyroid hormones during gestation (Pop et al., 2018) and troponin levels after cardiac surgery (Deneer et al., 2020). The application of omics technologies, such as transcriptomics and metabolomics, to study the dynamics of biological systems results in high-dimensional time-series data, in which the number of gene expression values or small molecules detected in biological fluids is larger than the number of samples. Statistical analysis of high-dimensional time-series data is challenging. Mechanistic modeling offers a complementary approach to study the dynamics of biological systems (van Riel, 2006). Differential equation models can be used to describe disease progression. For example, the model by de Winter et al. (2006) is composed of three differential equations to simulate glucose, insulin and HbA1c (glycated hemoglobin) over time in patients with diabetes. Dynamic metabolic models calibrated to time-series data have been developed for biological systems such as yeast (e.g., Rizzi et al., 1997; van Riel et al., 1998) and human metabolism (e.g., Rozendaal et al., 2018a; O'Donovan et al., 2019). *In silico* dynamic models often lack the multi level layers of regulation that control metabolism. This impedes their application in disease modeling because causes of disease can be located at multiple levels, and also molecular therapies can be targeted to genes, proteins and metabolites. To overcome current limitations in statistical analysis and mechanistic modeling we combine metabolic modeling with machine learning techniques to integrate longitudinal metabolic and transcriptomic data. Previously we developed the computational approach called ADAPT (Analysis of Dynamic Adaptations in Parameter Trajectories) (Tiemann et al., 2011; van Riel et al., 2013). ADAPT combines mechanism-based differential equation models with machine learning to model temporal metabolic data (Tiemann et al., 2013; Rozendaal et al., 2018b). ADAPT functions as a so-called state observer (or state estimator), which is a system that provides an estimate of the internal state of a given real system from measurements of the input and output of the real system. Here, we aimed to extend ADAPT to include both metabolic and transcriptomic time-series data. Hereto we added a new regularization function to the learning algorithm that is used to estimate model parameters. The new version of ADAPT uses the metabolite data as input to estimate trajectories of metabolic parameters and takes the gene expression data as additional information to refine the trajectories.

ADAPT has been applied to a model of hepatic lipid and plasma lipoprotein metabolism (HepaLip2) to predict which metabolic adaptations are induced upon pharmacological treatment of mice by Liver X receptor (LXR) agonist T0901317. LXR agonists exert potent antiatherosclerotic actions but simultaneously induce excessive triglyceride (TG) accumulation in the liver. Using the new version of ADAPT we reveal that both input and output fluxes to hepatic TG content are considerably induced on LXR activation and that in the early phase of LXR agonism, hepatic steatosis results from only a minor imbalance between the two. It is generally believed that LXR-induced hepatic steatosis results from increased *de novo* lipogenesis (DNL). In contrast, ADAPT predicts that the hepatic influx of free fatty acids is the major contributor to hepatic TG accumulation in the early phase of LXR activation. This prediction is tested *in vivo* by a metabolic tracer experiment.

## 2. Results

### 2.1. HepaLip2: Model of Hepatic Lipid and Plasma Lipoprotein Metabolism

**Figure 1:**
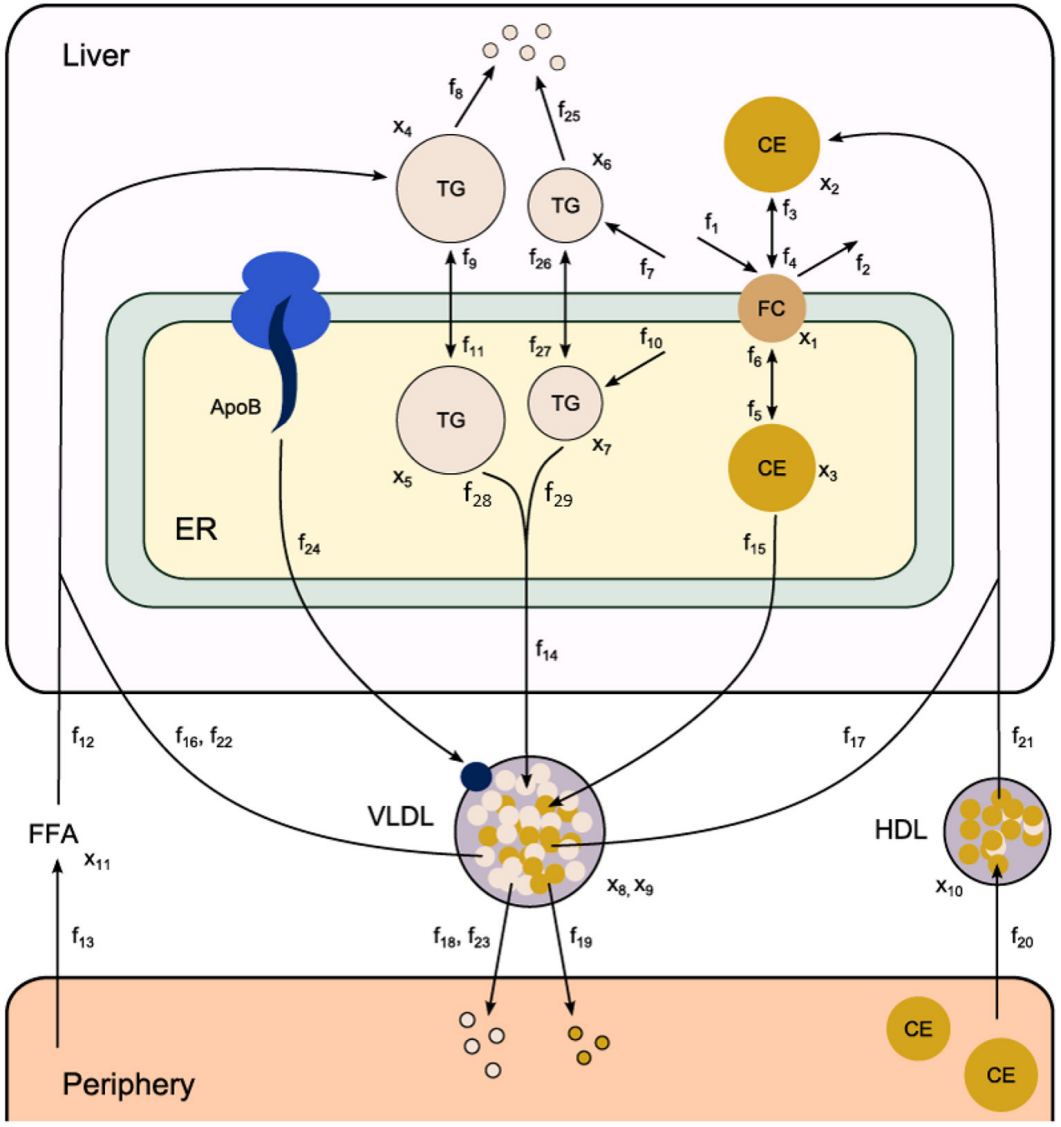
Computational model of hepatic lipid and plasma lipoprotein metabolism (HepaLip2), under fasting conditions. The HepaLip2 model is composed of three compartments representing the liver cytosol, liver endoplasmic reticulum, and blood plasma. The liver compartment includes reactions comprising the production, utilization, and storage of triglycerides and cholesterols, and the mobilization of these metabolites to the endoplasmic reticulum, where they are incorporated into nascent VLDL particles. The VLDL particles are secreted in the plasma compartment where they serve as energy source for peripheral tissues. Remnant particles are taken up and cleared by the liver. The model furthermore includes the hepatic uptake of free fatty acids as well as HDL-mediated reverse cholesterol transport. The model is composed of 11 differential equations (and 11 corresponding state variables *x*), 29 fluxes *f* and 22 (unknown) parameters. ApoB, apolipoprotein B; CE, cholesterylester; ER, endoplasmic reticulum; FFA, free fatty acid; FC, free cholesterol; HDL, high density lipoprotein; TG, triglyceride; VLDL, very low density lipoprotein.

Fundamental in ADAPT is a mathematical model of the (molecular) pathways of interest. We developed a mathematical multi-compartment model describing triglyceride and cholesterol metabolism (*HepaLip*2). The mathematical model contains three compartments representing the liver cytosol, liver endoplasmic reticulum (ER) and blood plasma (Figure 1). The liver includes the production, utilization and storage of triglycerides (TG) and cholesterols. Triglycerides are produced in the ER and can be transferred to the cytosol where they are stored in lipid droplets or catabolized. TG produced in the ER are also incorporated into nascent produced very low density lipoprotein (VLDL) particles. These VLDL particles are subsequently secreted in the blood plasma where they provide nutrients for peripheral tissues. The model also includes the hepatic uptake of free fatty acids (FFA) from plasma that predominantly originate from adipose tissue. Finally, the model includes the reverse cholesterol transport pathway, i.e., the net transport of cholesterol from peripheral tissues back to the liver via high density lipoproteins (HDL). The model is composed of 11 differential equations, (Table 1) 29 fluxes and 22 parameters. The flux equations are based on mass-action kinetics. Each flux equation introduces a parameter with unknown *in vivo* value. Collectively these parameters are referred to as the 'metabolic parameters'. A detailed description of the mathematical model including an overview of the state variables, parameters, fluxes, and differential equations is presented in the Supplementary Material (section 2).

**Table 1:** State variables included in the HepaLip2 model (Figure 1).

**State**	**Name**	**Description**	**Units**
*x*_1_	*x*_*FC*_	Hepatic free cholesterol	μ mol/liver
*x*_2_	*x*_*CE*_*cyt*__	Hepatic cholesteryl ester (cytosol)	μ mol/liver
*x*_3_	*x*_*CE*_*ER*__	Hepatic cholesteryl ester (ER)	μ mol/liver
*x*_4_	*x*_*TG*_*cyt*__	Hepatic triglyceride (cytotol)	μ mol/liver
*x*_5_	*x*_*TG*_*ER*__	Hepatic triglyceride (ER)	μ mol/liver
*x*_6_	*x*_*TGdnl*_*cyt*__	Hepatic *de novo* triglyceride (cytosol)	μ mol/liver
*x*_7_	*x*_*TGdnl*_*ER*__	Hepatic *de novo* triglyceride (ER)	μ mol/liver
*x*_8_	*x*_*TG*_*VLDL*__	Plasma VLDL-triglyceride	μ mol/L
*x*_9_	*x*_*C*_*VLDL*__	Plasma VLDL-cholesterol	μ mol/L
*x*_10_	*x*_*C*_*HDL*__	Plasma HDL-cholesterol	μ mol/L
*x*_11_	*x*_*FFA*_	Plasma free fatty acid	μ mol/L

*The differential equations, parameters and fluxes are presented in Supplementary Material (section 2)*.

### 2.2. Pharmacological Treatment With LXR Agonists

The liver X receptor (LXR) plays a central role in the control of cellular lipid and cholesterol metabolism and is considered a potential target to treat or prevent atherosclerosis. However, a serious complication of LXR activation is the excessive accumulation of triglycerides in the liver, which finally results in the development of hepatic steatosis. The underlying molecular mechanisms inducing these adaptations are not fully understood, which complicates the clinical application of LXR agonists (Grefhorst et al., 2002; Grefhorst and Parks, 2009; Cave et al., 2016). We used data obtained from pharmacological treatment of mice by LXR agonist T0901317 up to 3 weeks. Quantitative experimental data at different stages of the treatment intervention were collected to study the dynamics of induced molecular adaptations. All the experiments were performed in the fasting state. Details about the experimental procedures can be found in section 5.

**Figure 2:**
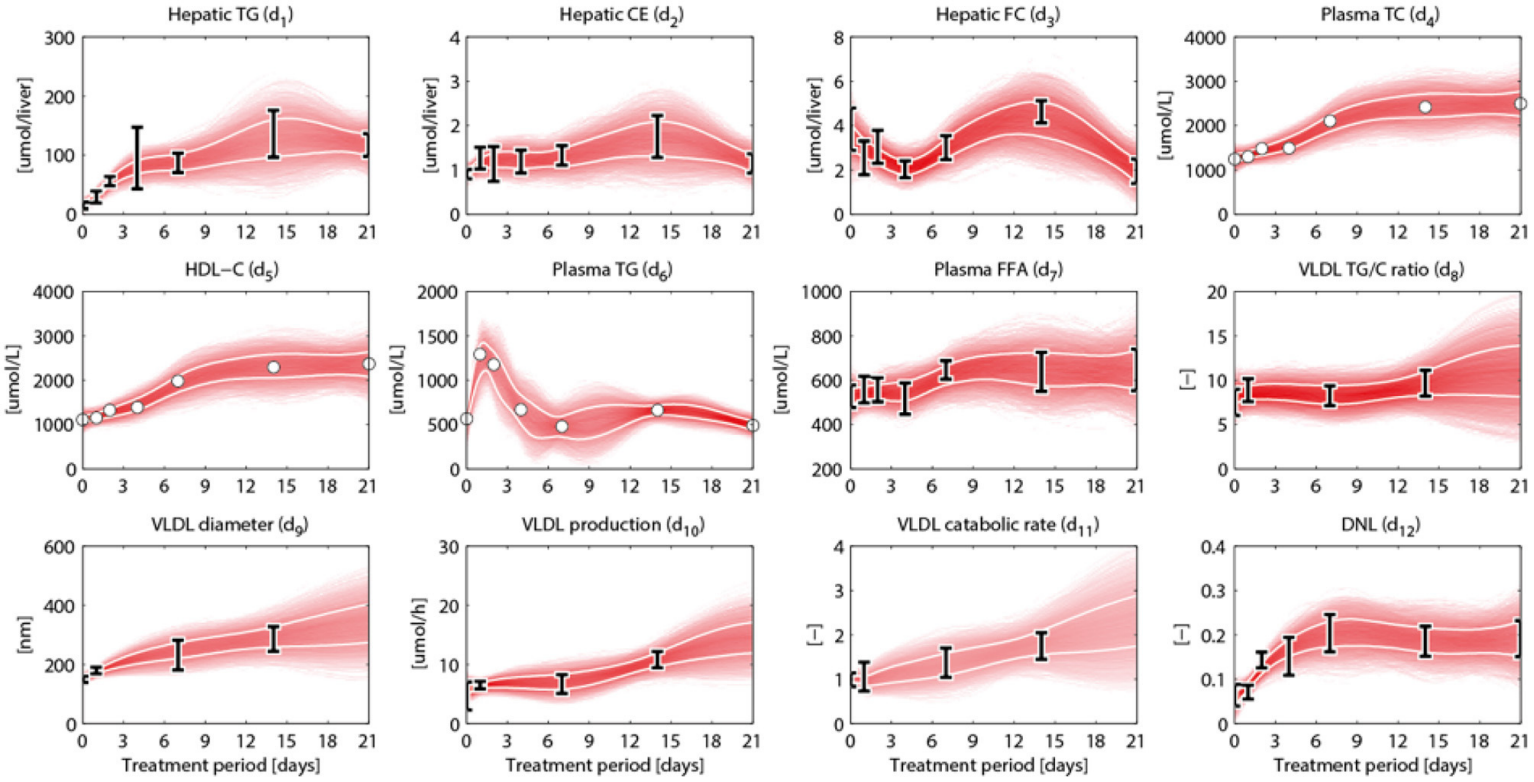
Metabolic data and interpolants. Metabolic time-series data and 2D histograms of the splines that were used as input for ADAPT (included in $\chi_{d}^{2}$, Equation 2). A darker color represents a higher density of trajectories in that specific region and time point. The white lines enclose the central 67% of the interpolant density at each time point. Data is represented as means ± standard deviations (*N* = 5–6), with an exception for the experimental data obtained via FPLC measurements. These measurements were performed on pooled mice plasma and are represented by the white dots. Measures of variance used for the Monte Carlo sampling of these quantities were estimated based on similar experiments that were performed in Grefhorst et al. (2012).

An overview of the quantities that were experimentally observed and their relation to corresponding model components is presented in Table 2. A model output *y*_*i*_ (*i* = 1, …, 15) was coupled to experimental data *d*_*i*_. Some model outputs are equal to state variables, other outputs are a combination (summation) of state variables. The data also includes fluxes, such as the synthesis rate of triglycerides secreted in VLDL particles, and the size and composition of VLDL particles and the corresponding variables in the model were also selected as outputs. Data was collected at 0, 1, 2, 4, 7, 14, and 21 days of treatment with T0901317 (Figure 2). Most measurements were available for all the seven time points, but *y*_13_ to *y*_15_ were experimentally observed for the untreated phenotype (*t* = 0) only.

**Table 2:** Measured quantities and their relation to model components.

**Measurement**	**Model output**	**Equation**	**Unit**
Hepatic triglyceride	*y*_1_	[*x*_4_] + [*x*_5_] + [*x*_6_] + [*x*_7_]	μ mol/liver
Hepatic cholesteryl ester	*y*_2_	[*x*_2_] + [*x*_3_]	μ mol/liver
Hepatic free cholesterol	*y*_3_	[*x*_1_]	μ mol/liver
Plasma total cholesterol	*y*_4_	[*x*_9_] + [*x*_10_]	μ mol/L
HDL-cholesterol	*y*_5_	[*x*_10_]	μ mol/L
Plasma triglyceride	*y*_6_	[*x*_8_]	μ mol/L
Plasma free fatty acid	*y*_7_	[*x*_11_]	μ mol/L
VLDL TG/C ratio	*y*_8_	*TG*_*cnt*_/*CE*_*cnt*_	[-]
VLDL diameter	*y*_9_	*D*_*VLDL*_	nm
VLDL-TG production	*y*_10_	*f*_14_	μ mol/h
VLDL catabolic rate	*y*_11_	*CR*_*VLDL*_	[-]
*de novo* lipogenesis	*y*_12_	*FC*_*DNL*_	[-]
Hepatic HDL-C uptake	*y*_13_^*^	*f*_21_	μ mol/h
Ratio cyt-TG / ER-TG concentration	*y*_14_^*^	*R*_*cTGcyt, TGer*_	[-]
Ratio cyt-TG / ER-TG production	*y*_15_^*^	*R*_*pTGcyt, TGer*_	[-]

* *Only for the untreated phenotype (t = 0)*.

### 2.3. Calibrating the Model to the Untreated Phenotype

First the HepaLip2 model was used to describe the untreated phenotype. Model parameters at baseline (start of simulation and experiment) are estimated from metabolic data and flux information. ADAPT estimates the model parameters by applying a least squares algorithm that minimizes the sum of squared errors (SSE) between the metabolic data *d*_*m,i*_ of the untreated phenotype and corresponding model outputs *y*_*i*_. To account for experimental and biological uncertainties different random samples of the data were generated assuming a data error model based on Gaussian distributions, with means and standard deviations according to the experimental data. A global scatter search was used to initialize a multi-start, gradient-based, interior point local optimization method, resulting in a collection of parameter sets that describe the untreated phenotype. These parameter sets served as a starting point from which ADAPT iteratively learns and updates the parameters to describe the transition between experimental data obtained during different stages of the treatment, as is described next.

### 2.4. Linking the Computational Model to Time-Series Data

HepaLip2 and ADAPT have been employed to generate insight in the LXR agonism response. The T0901317-induced perturbation starts at the proteome level and subsequently induces adaptations at the other levels. During the 3 week treatment the metabolic parameters and fluxes are expected to change over time. ADAPT captures adaptations or modulating effects on metabolic pathways by introducing time-dependent descriptions of model parameters. Parameter trajectories are constrained by experimental data. To enable the estimation of dynamic trajectories of metabolic parameters and fluxes, continuous dynamic descriptions of the experimental data are used as input for ADAPT. For this purpose, cubic smoothing splines were calculated that describe the experimental data, taking into account experimental and biological uncertainties. A collection of splines was calculated using a Monte Carlo approach as follows. For all time points in the data the same data model and sampling approach were used as described above for the untreated phenotype (the first time point in the time-series). Subsequently, for each generated set of time samples a cubic smoothing spline was fitted, which is used as input for the next step of the ADAPT algorithm. The experimental data and splines are presented in Figure 2.

### 2.5. Estimating Time-Dependent Changes of the Model Parameters

The HepaLip2 model mechanistically describes the kinetics of metabolic pathways (Figure 1). ADAPT is based on the assumption that during disease development and treatment response, changes in kinetic metabolic parameters are caused by changes in corresponding enzymes that catalyze conversion or transport of metabolites. Adaptations in metabolic processes are identified by inferring which metabolic parameters and consequently metabolic fluxes necessarily have to change to describe the experimental data. To this end, a simulation of the full treatment period was divided into a number *N*_*t*_ of time segments Δ*t*. First, the simulation is started using the parameters and model state of the untreated phenotype. Next, for each subsequent segment *n*, the system is simulated (using a variable step integration method) for a time-period Δ*t* using the parameters and model state of the previous step *n*−1 as a starting point. The parameters for segment *n* are re-estimated by minimizing the difference between the data interpolants and corresponding model outputs for that time segment. This procedure is repeated for all segments and as a result parameter trajectories are inferred by minimizing the objective function χ^2^ over the time segments through numerical optimization:

(1) $$\begin{array}{ll} \hat{\vec{p}}\left(n\Delta t\right)=\arg\underset{\vec{p}\left(n\Delta t\right)}{\min}\chi^{2}\left(\vec{p}\left(n\Delta t\right)\right) & n=1,\ldots ,N_{t} \end{array}$$

$\hat{\vec{p}}\left(n\Delta t\right)$ represents the optimized parameter set for the *n*th time segment. The objective function χ^2^ is the weighted sum of squared differences between model outputs and data:

(2) $$\begin{array}{l} \chi^{2}\left(\vec{p}\left(n\Delta t\right)\right)=\overset{N_{y}}{\underset{i=1}{\sum }}{\left(\frac{Y_{i}\left(n\Delta t\right)-d_{m,i}\left(n\Delta t\right)}{\sigma_{m,i}\left(n\Delta t\right)}\right)}^{2}≐\chi_{d}^{2}\left(\vec{p}\left(n\Delta t\right)\right) \end{array}$$

where *N*_*y*_ is the number of measured model variables (outputs), *Y*_*i*_(*n*Δ*t*) are the discrete time model outputs, *d*_*m,i*_(*n*Δ*t*) are the interpolants of the metabolic data with standard deviation σ_*m,i*_. The optimization procedure is repeated for all data interpolants, starting from the state and parameter set of the untreated phenotype. An ADAPT solution was considered acceptable if model outputs were within the 95% confidence interval of the data. In this study *N*_*y*_ = 15, and *N*_*t*_ = 200 was used.

ADAPT simulation of HepaLip2 provides estimates for system variables that were not experimentally observed, such as the synthesis rate and composition of VLDL particles (Supplementary Figure 7). As observed before (Tiemann et al., 2013), VLDL particle secretion is reduced upon LXR activation. Although the secretion of VLDL particles decreased, an increased release of VLDL-TG to the plasma was experimentally observed (Supplementary Figure 7B). Similarly, the computational analysis showed an increased production of VLDL-CE to the plasma (Supplementary Figure 7C). According to the model the progressive increase of these fluxes was facilitated by an increased loading of triglycerides and cholesterol onto VLDL particles (Supplementary Figures 7D,E). These predictions were obtained using only the metabolic data as input for ADAPT.

#### 2.5.1. Integration of Gene Expression Data

**Figure 3:**
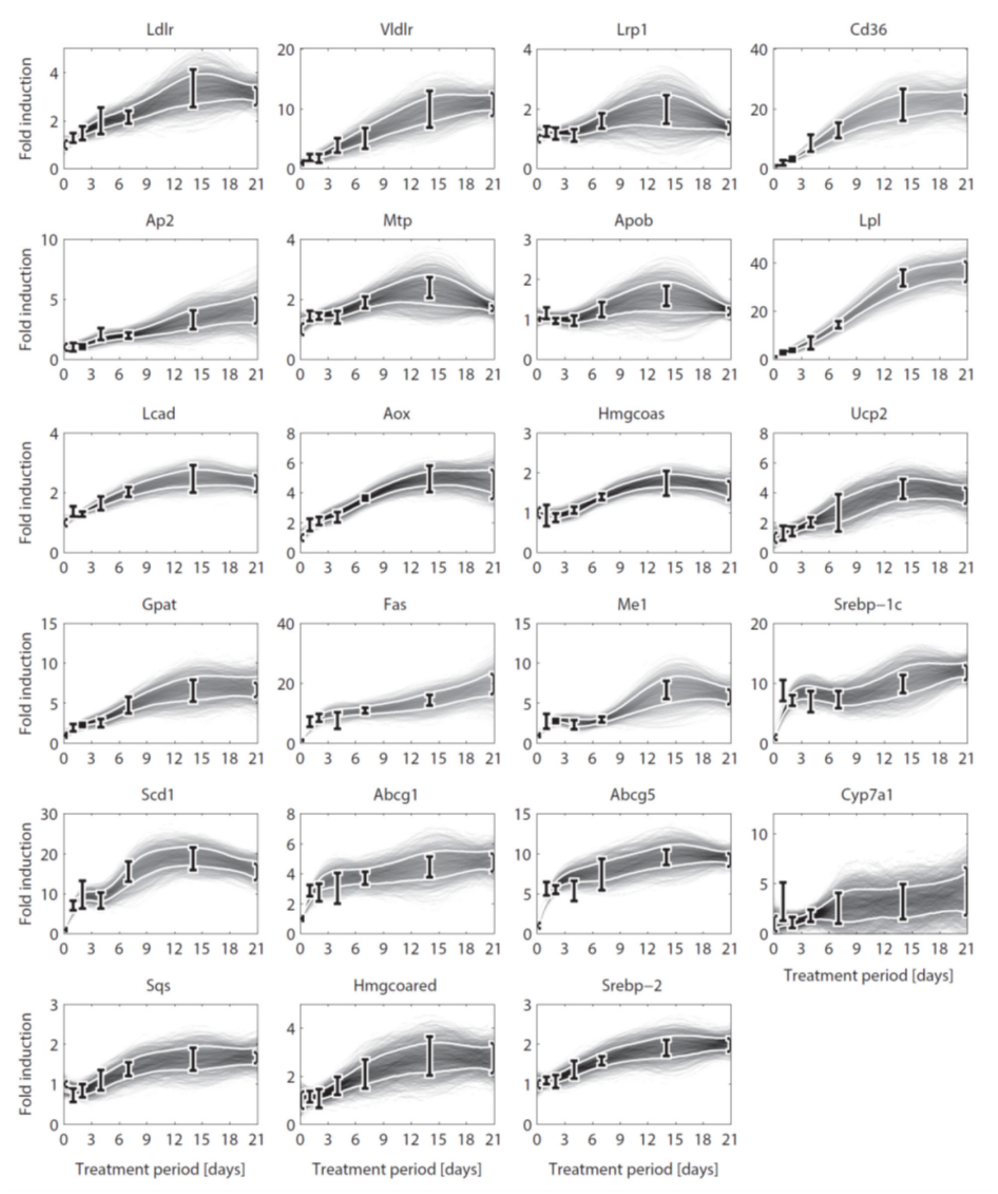
Gene expression data and interpolants. Temporal expression data for 23 genes and 2D histograms of the corresponding cubic splines that were used as input for ADAPT (included in $\chi_{g_{1}}^{2}$ and $\chi_{g_{2}}^{2}$). The experimental data is represented as means ± standard deviations (*N* = 5-6). The white lines enclose the central 67% of the interpolant density at each time point. (see Table 3 for the gene names).

Until here ADAPT connected metabolic parameters to activity of enzymes (protein level). Next, gene expression was added as a third layer of information. ADAPT has been extended to include a potential functional relationship between metabolic parameters and gene expression levels. Variables in the mechanistic (metabolic) part of the model can be directly linked to metabolic data, which is used to fit the model to that experimental data. Pathways at the transcriptome level were not modeled mechanistically due to the lack of sufficient quantitative information about these systems. Gene expression data does not have an one-to-one connection with the metabolic variables and, therefore, cannot be included in the error function (Equation 2). Therefore, a different approach was used to integrate gene expression data in the parameter trajectory estimation algorithm. The transcriptomic data is implicitly used to constrain the dynamic behavior of parameter trajectories, by including a regularization function. Time-course data of relative expression levels of 23 genes was available (Figure 3). Table 3 provides an overview of the parameters and genes that were coupled. The optimization problem was extended as follows. First, for each time segment Δ*t*, parameter adaptations are preferred such that resulting parameter trajectories and corresponding gene expression profiles display temporal correlation. This was implemented by including an additional component ($\chi_{g_{1}}^{2}$) in the objective function which maximizes the Pearson correlation between these profiles. Secondly, the gene expression data was also used to find parameter trajectories that are steady and smooth (enforcing temporal sparsity in the solutions). It was assumed that parameters are less likely to change when corresponding gene expression levels remain unchanged over time, compared to data indicating that expression of the genes is induced or repressed. Therefore, when transcriptomics data indicate that expression of genes changes over time parameter adaptations will be less penalized compared to genes with constant expression. This was implemented by including a third component ($\chi_{g_{2}}^{2}$) in the objective function which utilizes the time derivative of gene expression profiles to penalize parameter fluctuations. The higher the derivative of the gene expression profile, the lower the penalty on changes in parameter values will be. The resulting objective function $\chi^{2}\left(\vec{p}\right)$ is written as:

(3) $$\begin{array}{l} \chi^{2}\left(\vec{p}\right)=\chi_{d}^{2}\left(\vec{p}\right)+\lambda_{g_{1}}\chi_{g_{1}}^{2}\left(\vec{p}\right)+\lambda_{g_{2}}\chi_{g_{2}}^{2}\left(\vec{p}\right) \end{array}$$

in which $\chi_{d}^{2}$ is the (weighted) sum of squared errors (SSE) of metabolic data and model outputs (Equation 2), $\chi_{g_{1}}^{2}$ maximizes the temporal correlation between parameter trajectories and gene expression profiles, and $\chi_{g_{2}}^{2}$ penalizes parameter fluctuations. λ_*g*_1__ and λ_*g*_2__ are regularization constants (also referred to as weighting coefficients) that determine the relative importance of the components. Further details are provided in section 5 and in the Supplementary Material.

**Table 3:** Parameter-gene couples, linking 23 genes to 11 model parameters.

**Couple**	**Parameter**	**Gene**	**Description**
*c*_1, 1_	*p*_16_	Ldlr	Low-density lipoprotein receptor
*c*_1, 2_	*p*_16_	Vldlr	Very-low-density lipoprotein receptor
*c*_1, 3_	*p*_16_	Lrp1	Low-density lipoprotein receptor-related protein 1
*c*_2, 1_	*p*_12_	Cd36	Cluster of differentiation 36
*c*_2, 2_	*p*_12_	Ap2	Adipocyte protein 2
*c*_3, 1_	*p*_14_	Mtp	Microsomal triglyceride transfer protein
*c*_4, 1_	*p*_15_	Mtp	Microsomal triglyceride transfer protein
*c*_5, 1_	*p*_22_	Apob	Apolipoprotein B
*c*_6, 1_	*p*_18_	Lpl	Lipoprotein lipase
*c*_7, 1_	*p*_8_	Lcad	Long chain acyl-CoA dehydrogenase
*c*_7, 2_	*p*_8_	Aox	Aldehyde oxidase
*c*_7, 3_	*p*_8_	Hmgcoa	Hydroxymethylglutaryl-CoA
*c*_7, 4_	*p*_8_	Ucp2	Uncoupling protein 2
*c*_8, 1_	*p*_7_	Gpat	Glycerol-3-phosphate acyltransferase
*c*_8, 2_	*p*_7_	Fas	Fatty acid synthase
*c*_8, 3_	*p*_7_	Me1	NADP-dependent malic enzyme 1
*c*_8, 4_	*p*_7_	Srebp-1c	Sterol regulatory element binding transcription factor 1c
*c*_8, 5_	*p*_7_	Scd1	Stearoyl-CoA desaturase 1
*c*_9, 1_	*p*_10_	Gpat	Glycerol-3-phosphate acyltransferase
*c*_9, 2_	*p*_10_	Fas	Fatty acid synthase
*c*_9, 3_	*p*_10_	Me1	NADP-dependent malic enzyme 1
*c*_9, 4_	*p*_10_	Srebp-1c	Sterol regulatory element binding transcription factor 1c
*c*_9, 5_	*p*_10_	Scd1	Stearoyl-CoA desaturase 1
*c*_10, 1_	*p*_2_	Abcg1	ATP-binding cassette subfamily G member 1
*c*_10, 2_	*p*_2_	Abcg5	ATP-binding cassette subfamily G member 5
*c*_10, 3_	*p*_2_	Cyp7a1	Cytochrome P450, family 7, subfamily A, polypeptide 1
*c*_11, 1_	*p*_1_	Sqs	Squalene synthase
*c*_11, 2_	*p*_1_	Hmgcoared	HMG-CoA reductase
*c*_11, 3_	*p*_1_	Srebp-2	Sterol regulatory element-binding protein 2

*A description of the parameters and corresponding fluxes is presented in Supplementary Material (section 2)*.

### 2.6. Setting the Regularization Constants

In multi-objective optimization and regularized regression approaches, like Equation (3), the weights of the different components in the objective function are important hyper-parameters of the algorithm that are problem dependent and need to be tuned for adequate performance. First, the influence of the regularization constants for gene correlation (λ_*g*_1__) and damping of unnecessary parameter fluctuations (λ_*g*_2__) on the estimation of the parameter trajectories was investigated using a Monte Carlo approach. ADAPT was performed for 20, 000 random combinations for λ_*g*_1__ and λ_*g*_2__ and the values of the three components in the objective function were analyzed. Results are reported in the Supplementary Material (section 3.1). We found combinations of regularization constants for which $\lambda_{g_{1}}\chi_{g_{1}}^{2}$ becomes effective: when λ_*g*_1__ is larger than 10^−6^ and λ_*g*_2__ is smaller than 10^−8^ parameter-gene couples displayed temporal correlation. For these combinations λ_*g*_2__ is sufficiently large for $\lambda_{g_{2}}\chi_{g_{2}}^{2}$ to reduce unnecessary parameter trajectory fluctuations, and the data error $\chi_{d}^{2}$ is always small (Supplementary Figure 3).

Secondly, the characteristics of parameter trajectory solutions corresponding to a specific combination of gene regularization constants was investigated. In some cases parameter-gene couples already displayed (high) temporal correlation without including gene expression data (Supplementary Figure 4, bottom panel). As expected, in many cases an increase in temporal correlation between the assigned parameter-gene couples was obtained when gene expression data was included (Supplementary Figure 4, bottom panel). Interestingly, couple *c*_5,1_ showed a predominantly negative correlation for all solution groups. Couple *c*_5,1_ links the expression of *Apob* encoding for the apolipoprotein B to VLDL particle secretion (flux *f*_24_, parameter *p*_22_, Table 3). This can be explained when inspecting the VLDL particle secretion, described in detail in the Supplementary Material (section 3).

After these verification steps, we concluded the proposed method works as designed for Hepalip2 in combination with the experimental data: ADAPT provides a data-driven approach to incorporate the multi level layers of regulation in a dynamic model of metabolism. In the following sections we analyze the applicability of gene expression data to constrain model predictions, and ADAPT is applied to study: (1) the compartmentalization of hepatic triglycerides, (2) adaptations in the hepatic lipid loading capacity, and (3) the quantitative contribution of the different metabolic routes to the increased hepatic triglyceride level.

### 2.7. Integration of Gene Data Constrains Metabolic Predictions

**Figure 4:**
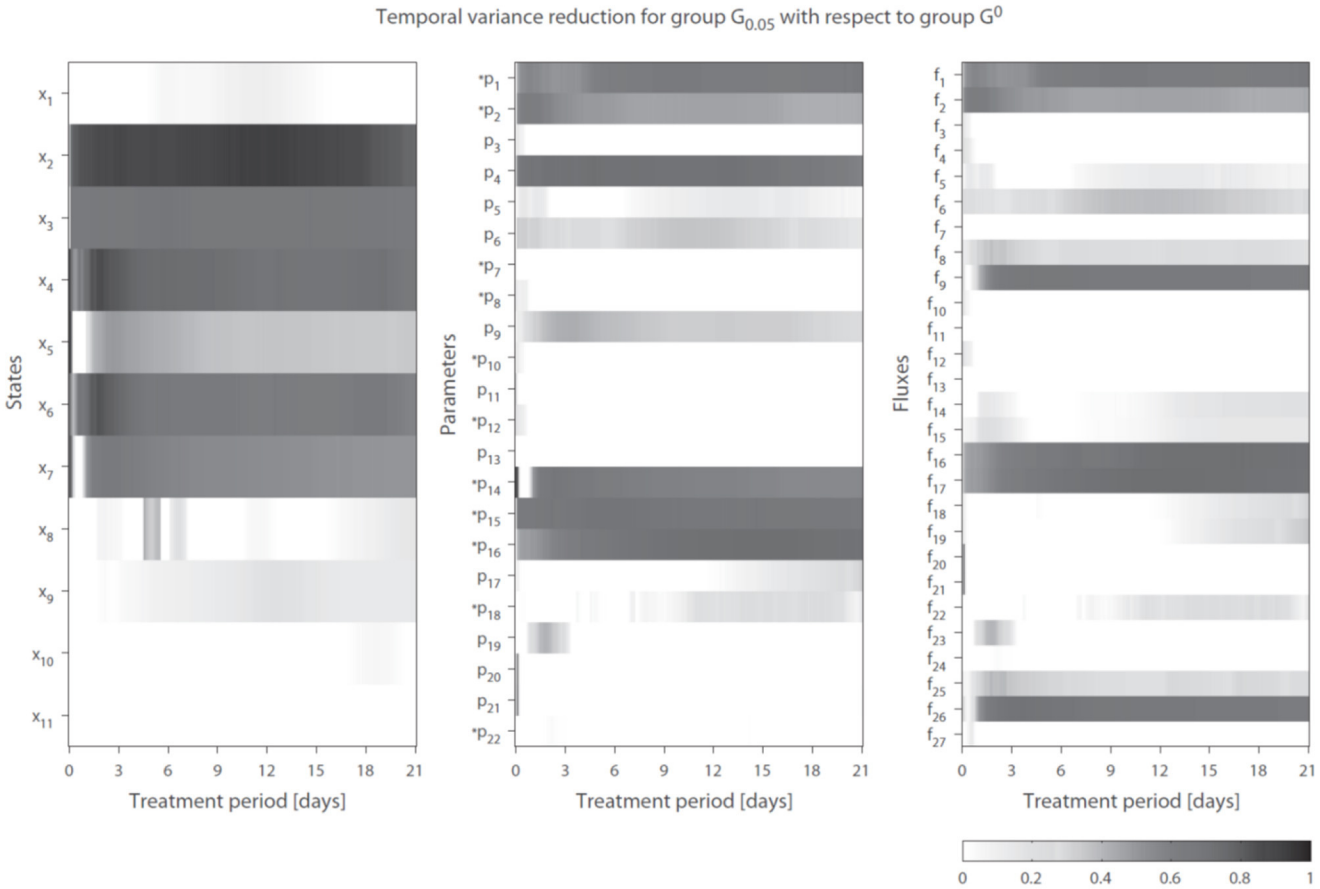
Temporal variance reduction by incorporating gene expression data. The gray-intensity indicates reduction in variance for estimated state variables **(left)**, parameters **(middle)**, and fluxes **(right)**. The asterisk signs (*) indicate parameters that were coupled to one or multiple genes. The (dark-)gray parts display model estimates that were effectively constrained by the gene expression data. Results shown for group *G*_0.05_, containing 5% of the trajectory solutions with the highest temporal correlation between parameter trajectories and gene expression (lowest penalty by $\chi_{g_{1}}^{2}$). Compared to G0, which are the solutions obtained without regularization Note *f*_14_ = *f*_28_ + *f*_29_.

We introduce the following notation: A group of trajectory solutions is denoted by *G*_*i*_ where *i* (0.05 ≤ *i* ≤ 1) represents the fraction of all solutions with the highest temporal correlations of parameter trajectories with gene expression over the entire treatment period (hence lowest $\chi_{g_{1}}^{2}$). For example, group *G*_0.05_ contains 5% of the 20, 000 trajectory solutions with the lowest values for $\chi_{g_{1}}^{2}$ summed over time. Furthermore, *G*^0^ is defined as the group of solutions corresponding to λ_*g*_1__ = λ_*g*_2__ = 0 (solutions obtained without regularization). The effect of integration of gene expression data on model performance was expressed as reduction in variance in model estimations (Equation 7 in the Supplementary Material). Figure 4 shows the variance reduction of *G*_0.05_ compared to *G*^0^ at each time point for all state variables (left panel), parameters (middle panel), and fluxes (right panel). The (dark-)gray parts clearly display model predictions that were effectively constrained by the gene expression data. Note that in multiple cases also a reduction in variance was obtained for parameters that were not coupled to genes.

### 2.8. Compartmentalization of Hepatic Triglycerides

**Figure 5:**
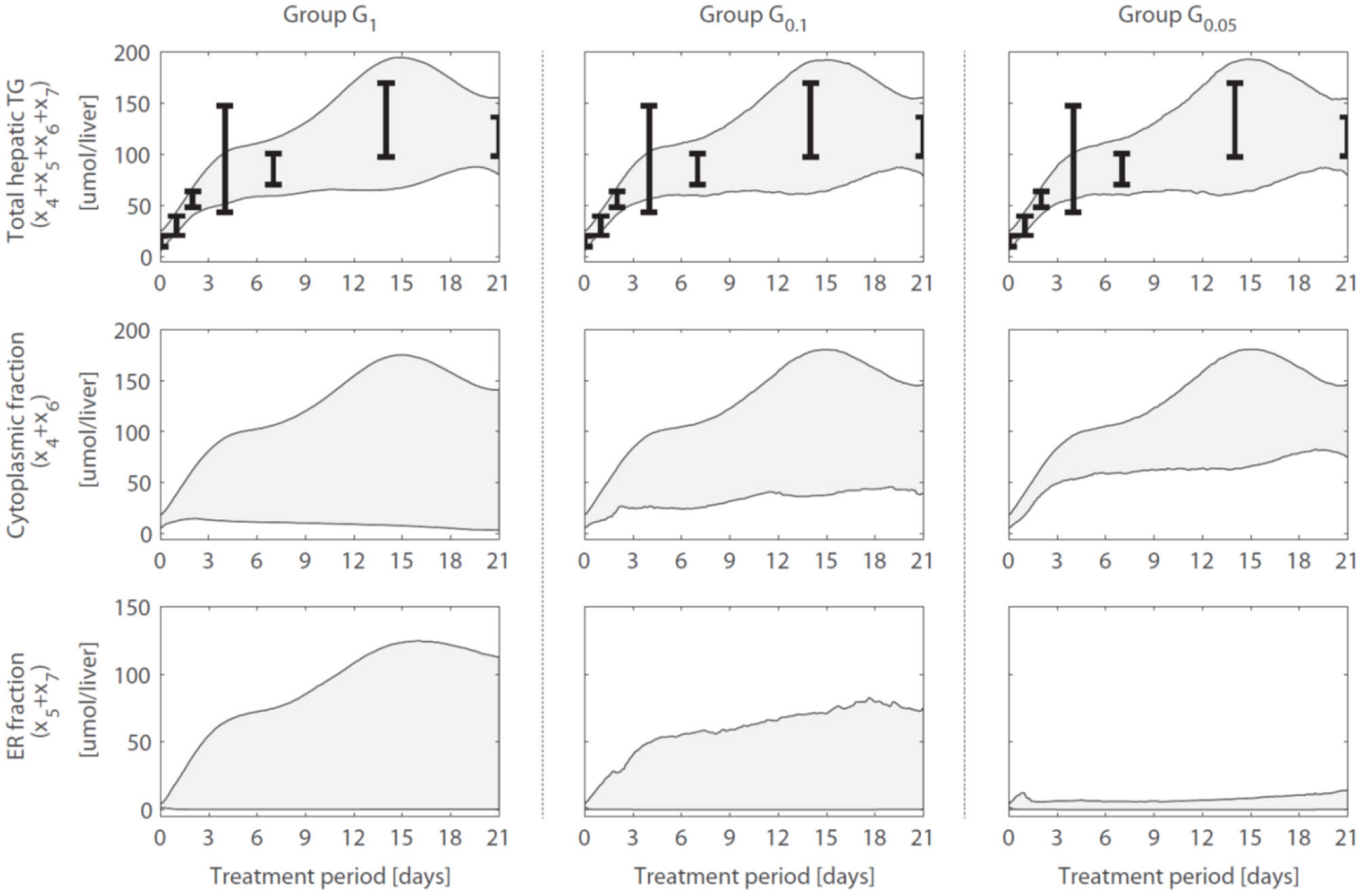
Hepatic triglyceride fluxes are increased and especially stored in the cytosolic fraction. Trajectories of the total hepatic TG content (*y*_1_ = *x*_4_ + *x*_5_ + *x*_6_ + *x*_7_), as well as its subdivision into cytosolic (*x*_4_ + *x*_6_) and endoplasmic reticulum (*x*_5_ + *x*_7_) fractions, are displayed for different groups of solutions. The experimental data of the total hepatic TG content (the error bars represent the standard deviation of the data) was included in the optimization procedure (linked to output *y*_1_) and all groups describe this data adequately. When only the metabolic data was used to calibrate the model (group *G*_1_), the distribution of the total TG content between the cytosolic fraction (TG in lipid droplets) and ER fraction (TG transferred to nascent VLDL could not be estimated precisely (left column). When including the gene expression data, model results showed that the increased TG pool is especially stored in the cytosol, rather than transferred to nascent VLDL (middle and right column). The solutions with the highest correlation between parameter trajectories and temporal gene expression (*G*_0.05_, right column) yielded the most precise estimates. The shaded areas indicate the 95% confidence intervals of the model estimates.

A reduction in the variance (estimation uncertainty) was observed for many of the model components when gene expression was included (Figure 4). One example concerns the hepatic storage of triglycerides in cytosolic (*x*_4_ + *x*_6_) and endoplasmic reticulum (*x*_5_ + *x*_7_) fractions. The cytosolic fraction represents the TG pool stored in lipid droplets and the ER fraction the TG contained in nascent VLDL particles. Figure 5 shows the 95% intervals of these model quantities for group *G*_1_ (Figure 5, left column), *G*_0.1_ (Figure 5, middle column), and *G*_0.05_ (Figure 5, right column). The experimental data only includes measurements of the total hepatic triglyceride content (*y*_1_) and the model provides more detailed information on where these lipids reside inside the hepatocyte. Experimental data of the total hepatic triglyceride content (*y*_1_ = *x*_4_ + *x*_5_ + *x*_6_ + *x*_7_) was included in the optimization procedure and all solution groups describe this data adequately. Before the inclusion of gene expression data, it was not possible to accurately predict how the total triglyceride content is distributed between cytosolic and VLDL fractions (Figure 5, left column). However, when the gene expression data was included, the model estimates that the increased triglyceride fluxes are especially stored in the cytosol, rather than transferred to nascent VLDL (Figure 5, middle and right column). This estimation was more precise for the 5% of the trajectory solutions with the lowest values for $\chi_{g_{1}}^{2}$ (highest temporal correlation with gene expression) compared to when the number of trajectories in the analysis was increased to include 10% of the trajectories with the lowest values for $\chi_{g_{1}}^{2}$ (*G*_0.05_ vs. *G*_0.1_).

**Figure 6:**
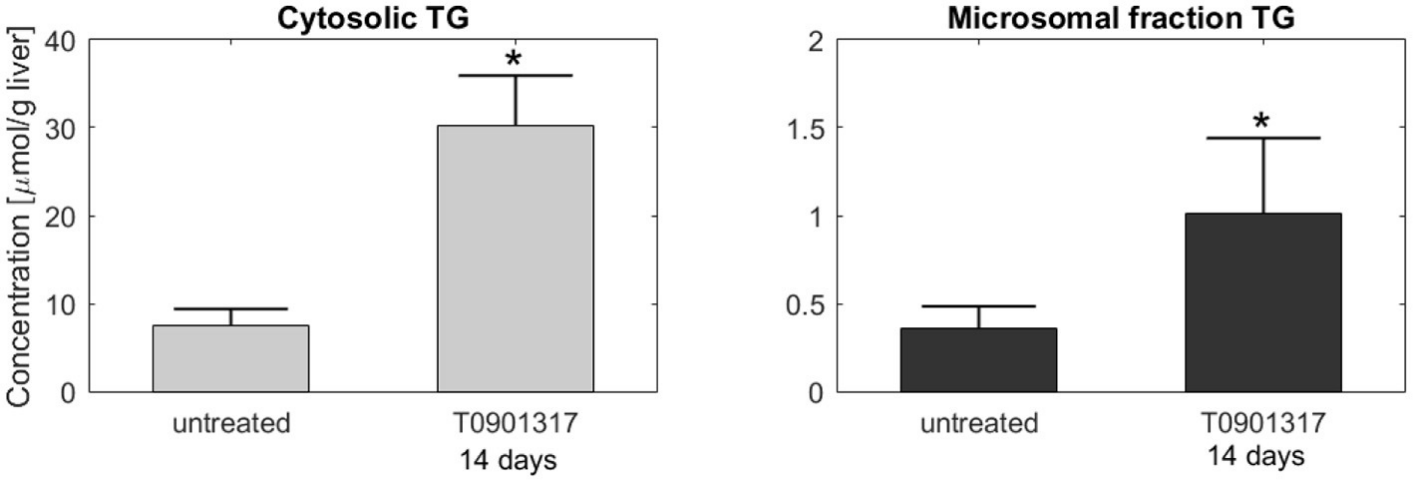
Fractionation of hepatic triglycerides. Additional measurements were performed on livers from C57BL/6J mice treated with T0901317 for 14 days and untreated controls to separate the cytosolic TG fraction from the microsomal fraction, containing VLDL particles. The experimental data shows that hepatic TG is predominantly stored in the cytosolic fraction, which confirmed the model estimations presented in Figure 5. Note the 20-fold scale difference in both y-axis. The bars indicate mean + standard deviation, *p < 0.05, Mann-Whitney *U*-test.

Subsequently, additional independent measurements were performed to validate this model result. Fractionation experiments were performed on livers from untreated C57BL/6J mice and C57BL/6J mice treated with T0901317 for 14 days, to separate the cytosolic triglyceride fraction from the microsomal fraction, containing VLDL. A description of the experimental materials and procedures is available in section 5. Indeed, the experimental data shows that the increased triglyceride fluxes are predominantly stored in the cytosolic fraction compared to the microsomal fraction (Figure 6), confirming the model prediction.

The parameter and flux trajectories were investigated to determine which processes are responsible for the observed compartmentalization of hepatic triglycerides between cytosolic and ER fractions (Supplementary Material, section 4). It appeared that the calculation of constrained estimations for the nascent VLDL triglyceride content was determined by two factors. First, the nascent VLDL triglyceride content is co-determined by the hepatic capacity to load these triglycerides onto nascent produced VLDL particles (fluxes *f*_14_ and *f*_15_). A second factor is the VLDL-TG production flux which increases progressively during the treatment (Supplementary Figure 7). Mathematically, this compartmentalization was enforced by a predicted increase of the hepatic lipid loading capacity of lipoproteins, as described before (Figure 5). The lipid loading capacity is co-determined by the activity of microsomal triglyceride transfer protein Mtp. Expression of Mtp is linked to parameters *p*_14_ and *p*_15_ in the HepaLip2 model. The expression level of the *Mtp* gene was increased upon LXR activation (Figure 3). Furthermore, a significant increase of the activity of Mtp was experimentally observed in mice treated with T0901317 for 1 week (Grefhorst and Parks, 2009).

### 2.9. Hepatic Triglyceride Accumulation

**Figure 7:**
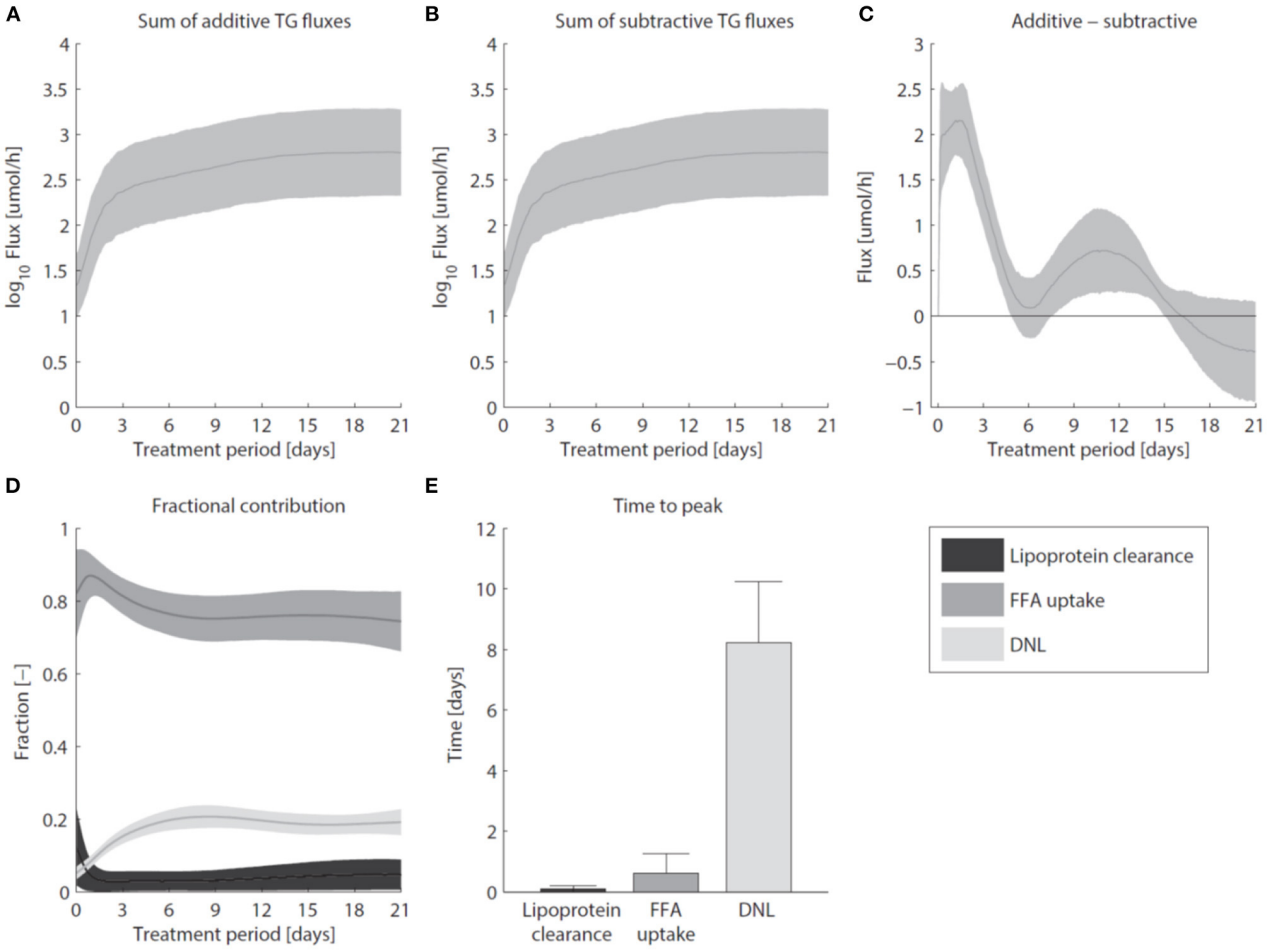
Hepatic accumulation of triglycerides. **(A)** The sum of fluxes contributing to the hepatic TG pool. **(B)** The sum of fluxes that catabolize hepatic TG. **(C)** The difference between additive and subtractive TG fluxes. Note the 10-fold smaller scale of the y-axis in **(C)** compared to **(A,B)**. **(D)** The fractional contribution of the various fluxes included in *F*_*a*_. **(E)** The time to peak (time to maximal fractional contribution) of the various processes. The areas and bars represent median ± median absolute deviation. The solutions of group *G*_0.05_ are displayed.

Pharmacological activation of LXR induces the excessive accumulation of triglycerides in the liver (Figure 7). Figure 7A shows that the sum of all fluxes contributing to the hepatic triglyceride pool increased rapidly during the first 3 days of the intervention, and remained at this elevated level upon prolonged treatment. In the mathematical model the additive fluxes (*F*_*a*_) include: *de novo* lipogenesis, hepatic FFA uptake from plasma, and clearance of lipoproteins via lipases and whole-particle uptake (Equation 8 in the Supplementary Material). Figure 7B shows that the increased *F*_*a*_ was closely accompanied by an increase of the fluxes that catabolize hepatic triglycerides (*F*_*s*_, Equation 9 in the Supplementary Material). The subtractive fluxes include the secretion of triglycerides to nascent produced VLDL particles and the hepatic catabolism of triglycerides (the hydrolysis of triglyceride into fatty acids and glycerol which are subsequently used in processes such as β-oxidation, gluconeogenesis, ketogenesis, sterol- and phospholipid synthesis). The difference between additive and subtractive triglyceride fluxes is displayed in Figure 7C. An imbalance between these fluxes can be observed during the first days of the intervention, which stabilizes gradually during the treatment. One process that contributes to the hepatic triglyceride accumulation is *de novo* lipogenesis. LXR induces the expression of lipogenic genes such as *Fas* (fatty acid synthase) and *Scd1* (stearoyl-CoA desaturase 1) (Figure 3), resulting in an increased fractional contribution of *de novo* lipogenesis (Figure 2). A question remained whether *de novo* lipogenesis is the sole process being responsible for the triglyceride accumulation. Experimental data and model simulations showed that the hepatic triglyceride level was already increased within 1 day of treatment, while no significant change in the fractional contribution of *de novo* lipogenesis was observed. This suggests that other processes are involved during the initial phase of the treatment (and perhaps also upon prolonged treatment). Therefore, we quantified the contribution of all metabolic routes included in the mathematical model that influence the hepatic triglyceride level. Figure 7D shows how the fractional contribution of the various fluxes included in *F*_*a*_ changes during treatment with T0901317. The analysis shows that plasma FFA provided a major contribution to the supply of hepatic triglycerides, whereas the clearance of lipoproteins played merely a minor role. Furthermore, the figure shows a peak contribution of hepatic FFA uptake at *t* ≈ 1 day, while the contribution of *de novo* lipogenesis increased gradually up to one week of treatment. Figure 7E shows the time to peak (time to maximal fractional contribution) of the various processes. The results clearly indicate that an increased uptake of FFA precedes the induction of *de novo* lipogenesis. The hepatic influx of FFA contributes for roughly 80% to the accumulation of TG in the liver.

**Figure 8:**
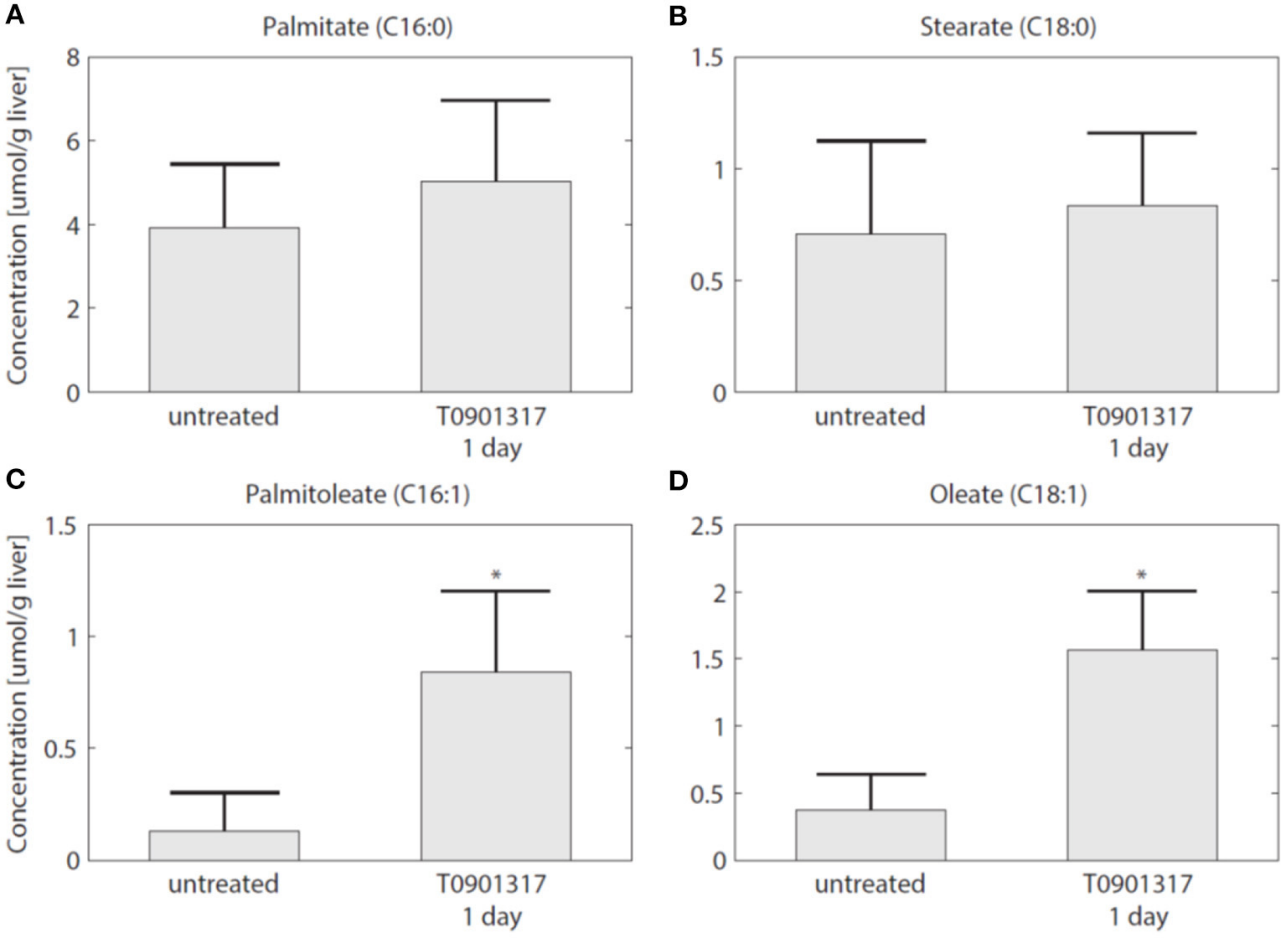
The hepatic uptake of FFA is increased. Additional experiments were performed in which ^13^C-palmitate was infused into C57Bl/6J mice that were treated with T0901317 for 1 day, and untreated controls. The different graphs present the contribution of plasma palmitate to hepatic palmitate **(A)**, stearate **(B)**, palmitoleate **(C)**, and oleate **(D)**. The contribution of plasma palmitate to hepatic palmitoleate and oleate was increased after 1 day of LXR activation, thereby confirming the model estimation presented in Figure 7. The bars represent mean + standard deviation, *p<0.05, Mann–Whitney *U*-test.

To establish whether the flux of FFA from plasma to the liver is indeed increased in the initial phase of LXR activation, as suggested by the model, experiments were performed in which ^13^C-palmitate was infused into C57Bl/6J mice that were treated with T0901317 for 1 day, and untreated controls (Hijmans et al., 2015). A description of the experimental materials and procedures is available in section 5. The contribution of plasma palmitate to hepatic palmitate and stearate were unchanged after 1 day of LXR activation (Figures 8A,B). However, LXR activation increased the flux from plasma palmitate to liver palmitoleate and oleate (Figures 8C,D), thereby confirming the model prediction obtained via ADAPT that FFA uptake increases within 1 day of treatment with T0901317.

## 3. Discussion

Biomedical applications of systems biology require to consider the complexity of the physiological system in humans or in the animals used to study human disease, including its highly interconnected structure and nonlinear dynamic behavior. The study of progressive adaptations during disease or intervention is complicated by the multilevel characteristics (metabolome, proteome, and transcriptome) of the underlying biological systems and the timescales on which these occur (seconds to years). Physiological parameters with diagnostic value are hidden in complicated, multivariate datasets. Time-series measurements of the metabolome provide information-rich data about the status of a biological system (Smilde et al., 2010). Gene expression data is abundant in literature and online repositories. However, it is not trivial to integrate multi-omics data, and hence to exploit the full potential of the information contained in these data. Multi-omics data is high-dimensional because the number of features and outcome variables is larger than the number of samples. Despite developments in machine/deep learning methods, data-driven approaches have fundamental limitations to model high-dimensional time series data. Mathematical modeling can construct computer simulation models from expert-based domain knowledge that can make transparent and explainable predictions of biological systems (mechanism-based systems biology models, van Riel, 2006). We proposed a combination of mathematical models and machine learning, implemented in ADAPT. ADAPT is less susceptible to data bias than data-driven, machine learning methods. Moreover, ADAPT quantifies uncertainty in the model and its predictions.

ADAPT is rooted in methods and techniques like system identification (from systems theory, Ljung, 1998), state-estimators (such as the Kalman filter, currently applied in navigation and positioning technology; Kalman, 1960) and data assimilation (in geosciences, such as weather forecasting, Asch et al., 2016). Characteristic is the use of a dynamical model of the system being analyzed in combination with statistical methods to incorporate measured data. Like a state-estimator, ADAPT combines dynamic models based on system knowledge with measurements and statistical models of uncertainties and variation in the process. The computer simulation model contains the elements and the dynamics of how the (complex) biological system operates. ADAPT connects the real biological system and the corresponding virtual model by different types of data, and the model updates (“learns”) as the biological counterparts changes. The algorithm requires time-series data to execute the model. It provides estimates for unobserved system variables and at time points for which data is not available. These state estimates are the “predictions” that can be made with ADAPT. In studies in humans and animals it is relatively easy to collect blood to perform measurements in. Via these measurements one often aims to get information about processes in organs and tissues. ADAPT enables the translation of plasma time-series metabolomics data to information about metabolic processes in tissues and between organs. In dedicated experiments with metabolic tracers and liver tissue was collected we have been able to validate estimates (predictions) of metabolic pools and fluxes to explain the development of hepatic steatosis as side-effect of treatment with a synthetic LXR agonist.

The application of advanced simulation models in (biomedical) systems biology and systems medicine requires credible models, that have been scrutinized on verification, validation and uncertainty quantification (Viceconti et al., in press). ADAPT addresses two major types of uncertainty in model estimation that impact model credibility and applicability: parametric uncertainty and structural uncertainty. First, *parametric uncertainty* concerns the problem of parameter identifiability. Values of model parameters are inferred from experimental data, but not all parameters might be identifiable from the available data (Raue et al., 2009; Vanlier et al., 2013). Since model parameters are estimated by calibrating the model to experimental data, uncertainty in the data (noise, errors) will propagate into the parameter estimates. Uncertainty in the parameter estimates subsequently will limit the accuracy of the model predictions. We used a stochastic data model from which samples were generated using a Monte Carlo approach. ADAPT was run for all samples hereby quantifying confidence in the estimated parameter trajectories. Parameter estimation in nonlinear dynamic models remains a computationally challenging task due to its non-convexity (presence of local optima) and ill-conditioning (Gábor and Banga, 2015). ADAPT uses a global scatter search to initialize a multi-start, gradient-based, interior point local optimization method. This approach was shown to be a successful strategy with a good performance in a benchmark study (Villaverde et al., 2019). A local solver (lsqnonlin in Matlab) is started from multiple start points to sample multiple basins of attraction associated with possible local minima in the cost function (the negative log-likelihood). The scatter search was made more efficient by only selecting the 10% of the most promising sampled parameters sets (lowest SSE) as start values for the local solver to estimate the model for the untreated condition (multistart with preselection).

Second, lack of knowledge about components and their quantitative interactions introduces uncertainty about the model structure. *Structural uncertainty* resides in simplifications that are inherent to the process of model building and assumptions that are made in case the nature and/or kinetic details of certain interactions are unknown (or disputed). The network topology of metabolic pathways is (relatively) well-known. Network structures impose strong constraints on the solution space of mathematical models, a characteristic that is employed in constraint-based simulation and analysis of (genome-scale) metabolic network models (Orth et al., 2010). Mathematical modeling of signal transduction and gene regulatory networks is more difficult. Insufficient information is available to formulate accurate mathematical descriptions of these networks. Making wrong and/or too strong assumptions about interactions and their kinetics could largely bias the model. Instead of adding equations with structural uncertainty, ADAPT introduces freedom in model parameters to compensate for unmodeled regulation.

ADAPT combines differential equation models of the network topology and mass fluxes in metabolic pathways with machine learning to model temporal metabolic data (Tiemann et al., 2013; Rozendaal et al., 2018b). A more complete understanding of underlying biological adaptations requires integration of other molecular data, such as transcriptomics and proteomics. Here we have extended ADAPT to integrate metabolic and transcriptomic time-series data. ADAPT uses numerical optimization for learning and updating of model parameters, to estimate the current state of the system and forecast its future trajectory. A new regularization function was added to the learning algorithm that is used to estimate model parameters. The new version of ADAPT uses the metabolite data as input to estimate trajectories of metabolic parameters and takes the gene expression data as additional information to refine the trajectories. The gene expression data was included implicitly in the model by incorporation in the regularization function (composed of two components $\chi_{g_{1}}^{2}$ and $\chi_{g_{2}}^{2}$), where it was implicitly used to guide and constrain the dynamic variations in the parameter trajectories. First, parameter adaptations were preferred such that resulting parameter trajectories and corresponding gene expression profiles display temporal correlation. Secondly, the gene expression data was used to prevent unnecessary (random) fluctuations in parameter trajectories, that could be the result of poor identifiability of certain parameters. The importance (weight) of each objective function component is determined by the corresponding regularization constant. The penalty function is a refinement of the regularization function described in Tiemann et al. (2013). $\chi_{g_{2}}^{2}$ effectuates that changing a parameter is costly, which will therefore be avoided unless it is required to describe the metabolic data. This results in parameter trajectories that are steady and smooth (enforcing temporal sparsity in the solutions). However, in the present study, the penalty of changing a parameter is reduced when corresponding gene expression level changes.

Regularization is a key component of ADAPT. It provides the possibility to extend the biological realism of the simulations by including post-transcriptional control that was not accounted for in the mathematical model. Regularization also improves numerical performance by resolving ill-conditioning of the estimation problem. Regularization is known to be beneficial for inverse problems, of which parameter estimation is an example. Regularized regression, like LASSO, is used to prevent overfitting and perform feature selection in computational statistics and machine learning (e.g., Imangaliyev et al., 2018). Regularization for estimating models of dynamical systems has been investigated in much lesser extent (Chen, 2013). We and others have shown that regularization can be very effective to mitigate ill-conditioning when estimating dynamic systems biology models (van Riel et al., 2013; Gábor and Banga, 2015). In ADAPT regularization is extended beyond so-called ridge regression (also known as Tikhonov regularization), in which the regularization function penalizes deviations of the parameter estimates from their reference (nominal) values or *a priori* defined target values (Cedersund and Roll, 2009; Dolejsch et al., 2019). Regularized estimations ensure a trade-off between bias and variance, reducing overfitting, and allowing the incorporation of prior knowledge in a systematic way.

Previously we had applied a model of hepatic lipid and plasma lipoprotein metabolism using an earlier version of ADAPT and discovered how pharmacological activation of LXR induced the reverse cholesterol pathway, but with counter-intuitive behavior of scavenger receptor class B1 (SR-B1), a receptor that facilitates the hepatic uptake of cholesterol from HDL particles (Tiemann et al., 2013). Here we have included gene expression data that was not available in the previous work to study the development of hepatic steatosis, which is a serious side effect of pharmacological activation of LXR. Results from the computational analysis showed that the additional integration of gene expression data effectively constrained and improved estimations (model predictions). of the hepatic storage of triglycerides in cytosolic and nascent VLDL fractions (Figure 5). Without the gene expression data it was not possible to accurately estimate how the total triglyceride content is distributed between these fractions. Interestingly, when the gene expression data was included, model predictions indicated that the increased triglyceride fluxes are predominantly stored in the cytosol, rather than being transferred to nascent VLDL. Hepatic fractionation experiments were subsequently performed that confirmed this prediction, providing an independent validation of the model.

As LXR induces the expression of lipogenic genes, such as *Fas* and *Scd1*, it was expected that *de novo* lipogenesis would be the major metabolic route contributing to development of hepatic steatosis. Experimental data shows that the hepatic triglyceride level was already increased within 1 day of treatment. The parameter and flux trajectories obtained with ADAPT were used to quantitatively analyze the contribution of all metabolic routes included in the mathematical model to the accumulation of hepatic triglycerides. Remarkably, the computational analysis revealed that plasma FFA provided a major contribution to the supply of hepatic triglycerides. Moreover, a peak contribution of hepatic FFA uptake was observed at 1 day of treatment, while the contribution of *de novo* lipogenesis increased gradually up to 1 week of treatment. The computational results clearly indicated that an increased uptake of FFA precedes the induction of *de novo* lipogenesis. This prediction was validated in an independent experiment with a metabolic tracer. To establish whether the flux of FFA from plasma to the liver is increased upon LXR activation, ^13^C-palmitate was infused via jugular vein catheter into C57Bl/6J mice that were treated with T0901317 for 1 day, and untreated controls. Indeed, an increased incorporation of ^13^C was observed in the hepatic triglyceride levels of palmitoleate and oleate confirming plasma as main source, as predicted by the model. Our findings might also be relevant to understand the development of steatosis, non-alcoholic fatty liver disease (NAFLD) and non-alcoholic steatohepatitis (NASH) associated with Metabolic Syndrome (Rozendaal et al., 2018b). Increased flux of FFA and glycerol from lipolysis of white adipose tissue (O'Donovan et al., 2019) has been associated with liver steatosis and NAFLD, also contributing to impaired postprandial repression of endogenous glucose production occurring in Type 2 Diabetes (Perry et al., 2015; Roden and Shulman, 2019).

ADAPT can be used to extract information on disease development and effects of medication that cannot be directly observed from the data. The computational model functions as a state-estimator applied to monitor the effect of therapeutic interventions and detect critical transitions of the system. Future developments include applications in so-called digital twinning in which computer simulation models are connected to their biological counterparts by different types of data and the model automatically updates as the biological counterpart changes (van Riel et al., 2020).

## 4. Conclusions

The development of computational models and techniques to study molecular adaptations during disease or intervention are important challenges in the field of biomedical systems biology and systems medicine. ADAPT combines the data-driven power of machine learning with that of knowledge-based, mechanistic simulation models. We presented an extension of ADAPT to integrate metabolomic and transcriptomic time-series data using a novel regularization approach. The gene expression data effectively constrained and improved model predictions, providing new insights in triglyceride metabolism related to drug-induced development of hepatic steatosis.

## 5. Materials and Methods

The computational workflow of ADAPT is described. First, the mathematical modeling of metabolic pathways and the identification of molecular adaptations are discussed. Second, the methodology to integrate gene expression data is presented.

### 5.1. Continuous Descriptions of the Experimental Data

**Figure 9:**
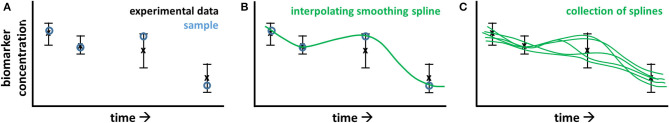
Pre-processing of experimental data for ADAPT. The experimental data consists of time course, longitudinal data obtained at multiple, discrete points in time, describing the transition of the biological system. In **(A)**, the black error bars represent mean and standard deviation of the data at each point in time. A time continuous description of the data is obtained by spline interpolation. To account for experimental and biological uncertainties, a Monte Carlo approach is used. The data is randomly sampled assuming a data error model based on Gaussian distributions with means and standard deviations according to the experimental data (**A**; blue circles). A cubic smoothing spline (**B**; green line) is fitted through these samples. This process is repeated, obtaining a collection of splines **(C)**.

Progressive diseases affect multiple processes operating at different levels (metabolome, proteome, and transcriptome) and different timescales (seconds to years). During disease development metabolic parameters (and consequently metabolic fluxes and concentrations) can be expected to change over time. The concept of time-dependent model parameters is introduced to study these adaptations. ADAPT identifies necessary dynamic changes in the model parameters to describe the transition between experimental data obtained during different stages (time points) of the disease. To estimate dynamic trajectories of model parameters, continuous dynamic descriptions of the experimental data were used as input for ADAPT. Cubic smoothing splines were calculated to describe the dynamics of the experimental data. To account for experimental variance and biological variation a collection of splines was calculated using a Monte Carlo approach. Different random samples of the experimental data were generated assuming Gaussian distributions with means and standard deviations according to the data. Subsequently, for each generated sample a cubic smoothing spline was calculated (Figure 9).

In the present study, a distinction between two types of data was made. First, metabolic data was acquired, e.g., concentrations and fluxes of metabolites in plasma and tissue compartments. The splines describing this data are denoted by ${\vec{d}}_{m}\left(t\right)$. Secondly, experimental data derived from the transcriptome level was considered, e.g., mRNA expression levels of genes. Corresponding splines are denoted by ${\vec{d}}_{t}\left(t\right)$.

### 5.2. Mathematical Modeling of the Metabolome Level

Mathematical modeling was centered on metabolic pathways. Pathways at the proteome and transcriptome levels that modulate the metabolic processes were not modeled explicitly as insufficient information of the underlying network structure and interaction mechanisms was available. The metabolic model is defined by a set of (non)linear ordinary differential equations (state-space structure):

(4) $$\begin{array}{l} \begin{array}{c} \overset{∙}{\vec{x}}\left(t\right)=\text{N}\vec{f}\left(\vec{x}\left(t\right),\vec{p},\vec{u}\right)\;\text{with}\;\vec{x}\left(t_{0}\right)={\vec{x}}_{0}\\ \vec{y}\left(t\right)=\vec{g}\left(\vec{x}\left(t\right),\vec{p},\vec{u}\right) \end{array} \end{array}$$

where $\overset{∙}{\vec{x}}$ is a vector of first derivatives of molecular species (or state variables) $\vec{x}$ with respect to time. The right-hand side of the equation is given by the topology of the network (stoichiometric matrix **N**) and a set of functions $\vec{f}$ that describe the interactions between the species. The initial concentrations of $\vec{x}$ are given by ${\vec{x}}_{0}$. The vector $\vec{y}$ represents the model outputs, which are given by a set of functions $\vec{g}$ that map the model states to specific quantities of interest. The outputs usually are quantities that have been experimentally measured. Both functions $\vec{f}$ and $\vec{g}$ depend on kinetic parameters $\vec{p}$ and optional external inputs $\vec{u}$. In principle, the generic set of equations in (4) can be used to describe any biomolecular reaction network. Here we use the system of ordinary differential equations to describe metabolic networks.

### 5.3. Dynamic Parameters to Describe Metabolic Adaptations

Details of the ADAPT methodology have been described in Tiemann et al. (2013) and are repeated here briefly for consistency. Dynamic adaptations in metabolic processes are identified by inferring necessary dynamic changes in the model parameters which are therefore time-dependent. To this end, a simulation of the treatment was divided in *N*_*t*_ steps of Δ*t* time period using the following discretization:

(5) $$\begin{array}{l} \begin{array}{c} \vec{X}\left(n\Delta t\right)=\vec{x}\left(\Delta t,\vec{p}\left(n\Delta t\right)\right)\\ \vec{Y}\left(n\Delta t\right)=\vec{g}\left(\vec{X}\left(n\Delta t\right),\vec{p}\left(n\Delta t\right)\right)\\ \;\vec{X}\left(0\right)={\vec{x}}_{0}\left(\vec{p}\left(0\right)\right) \end{array} \end{array}$$

with 0 ≤ *n* ≤ *N*_*t*_ and *N*_*t*_Δ*t* the time period of the entire experiment. The simulation is initiated (*n* = 0) using the initial values of the model states ${\vec{x}}_{0}$ obtained with parameter set $\vec{p}\left(0\right)$ representing the untreated phenotype. Subsequently, for each step *n* > 0 the system is simulated for a time period of Δ*t* using the final values of the model states of the previous step *n* − 1 as initial conditions. Parameters $\vec{p}\left(n\Delta t\right)$ are estimated by minimizing the difference between the data interpolants $\vec{d_{m}}\left(n\Delta t\right)$ and corresponding model outputs $\vec{Y}\left(n\Delta t\right)$. Here, the previously estimated parameter set $\vec{p}\left(\left(n-1\right)\Delta t\right)$ is provided as initial set for the optimization algorithm. The parameter optimization problem is given by:

(6) $$\begin{array}{l} \hat{\vec{p}}\left(n\Delta t\right)=\arg\underset{\vec{p}\left(n\Delta t\right)}{\min}\chi_{d}^{2}\left(\vec{p}\left(n\Delta t\right)\right) \end{array}$$

(7) $$\begin{array}{l} \chi_{d}^{2}\left(\vec{p}\left(n\Delta t\right)\right)=\overset{N_{y}}{\underset{i=1}{\sum }}{\left(\frac{Y_{i}\left(n\Delta t\right)-d_{m,i}\left(n\Delta t\right)}{\sigma_{m,i}\left(n\Delta t\right)}\right)}^{2} \end{array}$$

where $\hat{\vec{p}}\left(n\Delta t\right)$ represents the optimized parameter set and $\chi_{d}^{2}$ is the weighted sum of squared errors (SSE), with *N*_*y*_ the number of model outputs (equal to the number of measured variables). Parameter trajectories were estimated using 200 time steps (*N*_*t*_ = 200).

A Monte Carlo approach was employed to account for methodological and experimental uncertainties. First, a global scatter search was used to initialize a multi-start local optimization method (Tiemann et al., 2011). 2 × 10^5^ parameter vectors were sampled from a widely dispersed range of initial parameter values (10^−6^ to 10^6^). For each parameter vector $\chi_{d}^{2}{|}_{n=0}$ was computed (SSE at *t* = 0). 2 × 10^4^ (10%) of the best performing parameter sets (with lowest $\chi_{d}^{2}{|}_{n=0}$) were selected and used to initiate the optimization procedure and estimate $\hat{\vec{p}}\left(0\right)$, using a gradient-based, interior point local optimization method (lsqnonlin in Matlab). This resulted in a collection of parameter sets that describe the untreated phenotype. Secondly, in each optimization series a different spline function for ${\vec{d}}_{m}$ was used. Finally, distributions of parameter trajectories (and consequently state and flux trajectories) are obtained that describe the transition of the phenotype during the disease or intervention.

### 5.4. Implicit Integration of the Transcriptome Level

Time-course data of relative gene expression levels was used as an additional source of information to constrain the dynamic behavior of parameter trajectories. However, note that pathways at the transcriptome level were not modeled explicitly due to the lack of sufficient quantitative information about the gene regulatory networks regulating the response to LXR activation. Therefore, the parameter trajectory estimation protocol, as formulated in Equations (6) and (7), was modified to integrate gene expression data. ADAPT is based on the assumption that changes in metabolic parameters are reflected by changes in corresponding enzymes, which in turn are reflected by changes in corresponding gene expression levels. This implies there is a functional relationship between a metabolic parameter *p*_*i*_ and corresponding gene expression level *d*_*t,i*_.

#### 5.4.1. Maximization of the Temporal Correlation

The optimization problem presented in Equation (6) was extended as follows. For clarity we introduce the following definitions: $\vec{p}\left[\cdot n\right]=\vec{p}\left[0,\Delta t,\cdots n\Delta t\right]$ and ${\vec{d}}_{t}\left[\cdot n\right]={\vec{d}}_{t}\left[0,\Delta t,\cdots n\Delta t\right]$, which represents the parameter trajectories from time step 0 to *n* and corresponding gene expression data, respectively. During a re-optimization of the metabolic parameters $\vec{p}$ from step *n*−1 to step *n*, a $\Delta \vec{p}$ is preferred such that resulting parameter trajectories $\vec{p}\left[\cdot n\right]$ and corresponding gene expression profiles ${\vec{d}}_{t}\left[\cdot n\right]$ display temporal correlation. This was effectuated by including an additional component $\chi_{g_{1}}^{2}$ in the objective function which maximizes the temporal correlation between these profiles:

(8) $$\begin{array}{l} \chi_{g_{1}}^{2}\left(\vec{p}\left(n\Delta t\right)\right)=\overset{N_{p}}{\underset{i=1}{\sum }}V_{i}\left(n\Delta t\right) \end{array}$$

where *N*_*p*_ is the number of parameters, and *V*_*i*_(*n*Δ*t*) is given by:

(9) $$V_{i}(n\Delta t)=\{\begin{array}{ll} \frac{1}{N_{ci}}\sum_{j=1}^{N_{ci}}{\left(1-\rho_{ij}(n\Delta t)\right)}^{2} & \text{if }N_{ci}>0\\ 0 & \text{otherwise } \end{array}$$

where *N*_*ci*_ is the number of genes assigned to parameter *i*, and ρ_*ij*_(*n*Δ*t*) is given by:

(10) $$\begin{array}{l} \rho_{ij}\left(n\Delta t\right)=\frac{\text{Cov}\left({\vec{p}}_{i}\left[\cdot n\right],{\vec{d}}_{t,ij}\left[\cdot n\right]\right)}{\sigma \left({\vec{p}}_{i}\left[\cdot n\right]\right)\sigma \left({\vec{d}}_{t,ij}\left[\cdot n\right]\right)} \end{array}$$

Equation (10) represents the Pearson correlation coefficient between a parameter trajectory and corresponding gene expression data, which is bounded between −1 (maximal negative correlation) and 1 (maximal positive correlation). Note that multiple genes can be assigned to a parameter, which could be useful for instance when a cascade of molecular processes is integrated in a single mathematical reaction equation.

#### 5.4.2. Constraining the Magnitude of Dynamic Variations in Trajectories

The gene expression data was also used to constrain the magnitude of dynamic variations in the parameter trajectories. It was assumed that parameters are less likely to change when corresponding gene expression levels remain unchanged, compared to scenarios when expression of the genes is induced or repressed. Therefore, in latter cases parameter adaptations are less penalized compared to former cases. This was effectuated by including an additional objective function $\chi_{g_{2}}^{2}$ which utilizes the time derivative of gene expression profiles to penalize parameter fluctuations:

(11) $$\begin{array}{l} \chi_{g_{2}}^{2}\left(\vec{p}\left(n\Delta t\right)\right)=\overset{N_{p}}{\underset{i=1}{\sum }}W_{i}\left(n\Delta t\right) \end{array}$$

with *W*_*i*_(*n*Δ*t*) given by:

(12) $$W_{i}(n\Delta t)=\;\{\begin{array}{ll} \frac{1}{N_{ci}}\sum_{j=1}^{N_{ci}}{\left(\frac{P_{i}(n\Delta t)}{G_{ij}(n\Delta t)}\right)}^{2} & \text{if }N_{ci}>0\\ P_{i}(n\Delta t) & \text{otherwise} \end{array}$$

with *P*_*i*_(*n*Δ*t*) and *G*_*ij*_(*n*Δ*t*) defined as:

(13) $$\begin{array}{l} P_{i}\left(n\Delta t\right)=\frac{1}{p_{i}\left(0\right)}\frac{p_{i}\left(n\Delta t\right)-p_{i}\left(\left(n-1\right)\Delta t\right)}{\Delta t} \end{array}$$

(14) $$\begin{array}{c} G_{ij}(n\Delta t)={\frac{1}{d_{t,i,j}(0)}\frac{d}{dt}d_{t,i,j}(t)|}_{t=n\Delta t} \end{array}$$

where *P*_*i*_(*n*Δ*t*) represents the normalized derivative of parameter *i* at time step *n*. Relative derivatives were used to assign equal relevance to all parameters and to avoid domination of the optimization by large absolute values. Furthermore, *G*_*ij*_(*n*Δ*t*) represents the normalized derivative of the spline function (evaluated at time step *n*) that describes corresponding gene expression data. To avoid division by zero (when *G*_*ij*_(*n*Δ*t*) = 0), the minimal absolute value of *G*_*ij*_(*n*Δ*t*) was truncated at 10^−6^. Note that *P*_*i*_(*n*Δ*t*) effectuates that changing a parameter is costly, which will therefore be avoided unless it is required to describe the experimental data. However, when accompanied by a change in gene expression level, the penalty of changing corresponding parameter is reduced (because *P* is divided by *G*).

Objective functions $\chi_{g_{1}}^{2}$ and $\chi_{g_{2}}^{2}$ were formulated as soft constraints by introducing constants λ_*g*_1__ and λ_*g*_2__, which determine the contribution strengths of the functions. This implies that metabolic parameters and corresponding gene expression levels do not necessarily have to display temporal correlation when this is in contradiction to the metabolic data. This provides the possibility to account for post-transcriptional control. In summary, an optimized parameter set is defined as follows:

(15) $$\begin{array}{l} \hat{\vec{p}}(n\Delta t)=\underset{\vec{p}(n\Delta t)}{\text{arg min}}(\chi_{d}^{2}(\vec{p}(n\Delta t))+\lambda_{g_{1}}\chi_{g_{1}}^{2}(\vec{p}(n\Delta t))\\ \;+\lambda_{g_{2}}\chi_{g_{2}}^{2}(\vec{p}(n\Delta t))) \end{array}$$

The determination of the regularization constants is discussed in Supplementary Material (section 3.1).

### 5.5. Implementation Details

The mathematical model and ADAPT were implemented in MATLAB (The Mathworks, Natick, Massachusetts, USA). The code is available on GitHub (https://github.com/nvanriel/ADAPT, https://github.com/rcqsnel/adapt-modeling-framework, and https://github.com/yvonnerozendaal). The ordinary differential equations were solved with compiled MEX files using numerical integrators from the SUNDIALS CVode package (2.6.0, Lawrence Livermore National Laboratory, Livermore, California) (Hindmarsh et al., 2005). An absolute and relative tolerance of 10^−6^ were used. The MATLAB nonlinear least-squares solver lsqnonlin (from the Optimization Toolbox), which uses an interior reflective Newton method (Coleman and Li, 1996), was used to estimate model parameters. The termination tolerances for the objective function and the parameter estimates were set to 10^−10^, the maximum number of iterations allowed was set to 10^3^ and the maximum number of function evaluations allowed to 10^5^. Parameter trajectories were estimated using 200 time steps. The MATLAB function csaps (from the Curve Fitting Toolbox) was used to calculate cubic smoothing splines using the default smoothness setting (=1) and the roughness dependent on the variation in the data: (1/*std*)^2^ (std: standard deviation).

### 5.6. Experimental Procedures

The experimental procedures have been described previously (Tiemann et al., 2013; Hijmans et al., 2015). Information about the fractionation experiments is provided in the Supplementary Material. Experimental procedures were approved by the Ethics Committee for Animal Experiments of the University of Groningen.

## Data Availability Statement

All datasets generated for this study are included in the article/Supplementary Material.

## Ethics Statement

The animal study was reviewed and approved by Institutional Animal Care and Use Committee of the University of Groningen.

## Author Contributions

NR and AG conceived and designed the study. NR, AG, and PH supervised the research. CT developed the software and performed the simulations. CT and NR analyzed the results and wrote the paper. NR, PH, and AG read and revised the paper. All authors contributed to the article and approved the submitted version.

## Conflict of Interest

The authors declare that the research was conducted in the absence of any commercial or financial relationships that could be construed as a potential conflict of interest.

## Abbreviations

ADAPT: analysis of dynamic adaptations in parameter trajectories
apo: apolipoprotein
C: cholesterol
CE: cholesterylester
DNL: *de novo* lipogenesis
ER: endoplasmic reticulum
FC: free cholesterol
FFA: free fatty acid
FPLC: fast protein liquid chromatography
HDL: high density lipoprotein
LXR: liver X receptor
ODE: ordinary differential equation
SSE: sum of squared errors
TG: triglyceride
VLDL: very low density lipoprotein.
