# Supplementary material for: Metabolic Modeling Combined With Machine Learning Integrates Longitudinal Data and Identifies the Origin of LXR-Induced Hepatic Steatosis
Source: Front Bioeng Biotechnol. 2021 Feb 16;8:536957. doi: 10.3389/fbioe.2020.536957 (PMC7921164; doi:10.3389/fbioe.2020.536957)
Supplement: Supplementary file 1 [file Data_Sheet_1.pdf]

Supplementary Material

1 EXPERIMENTAL PROCEDURES

1.1 Subcellular fractionation

Livers were fractionated into cytosolic and microsomal membrane fractions essentially as described before (Chao et al., 1986). Briefly, freshly excised liver sections were homogenized (10% w/v) in ice-cold buffer (0.25M sucrose, 3mM Tris and 1mM EGTA, pH 7.4). Homogenates were spun at low speed (9,000 g) to remove cellular debris followed by high-speed centrifugation (105,000 g) to separate the cytosolic lipid fraction from the intact microsomal membrane fraction.

1.2 Statistics

Experimental data are presented as mean ± standard deviation. For nonparametric statistical analysis the Mann–Whitney U test was used.

2 HEPALIP2 MODEL

2.1 Description

A mathematical multi-compartment model, named HepaLip2, was constructed. The mathematical model contains three compartments representing the liver cytosol, liver endoplasmic reticulum and blood plasma. The liver includes the production, utilization and storage of triglycerides and cholesterol in lipid droplets, as well as the mobilization of these metabolites to the endoplasmic reticulum (ER) where they are incorporated into nascent very low density lipoprotein (VLDL) particles. These VLDL particles are subsequently secreted in the plasma compartment and provide nutrients for peripheral tissues. The model furthermore includes the hepatic uptake of free fatty acids (FFA) from the plasma that predominantly originate from adipose tissue. As FFA derived from triglycerides are oxidized in mitochondria, which are located in the cytosol, it was assumed that no oxidation takes place in the endoplasmic reticulum compartment (Gibbons et al., 2000). Finally, the model includes the reverse cholesterol transport pathway, *i.e.*, the net transport of cholesterol from peripheral tissues back to the liver via high density lipoproteins (HDL). Note that the transport of dietary lipids via chylomicrons was not included in the model, because the experiments were performed in the fasting state. A graphical representation of the model is provided in the main text (**Figure 1**).

2.2 Model equations

The mathematical model contains eleven metabolic species, which are the model state variables $\vec{x}$ (**Table 1** in the main text), interlinked by twenty-nine fluxes $\vec{f}$ (**Table S1**). The flux equations are based on mass-action kinetics, introducing twenty-two parameters $\vec{p}$. The model is written as a system of first order nonlinear ordinary differential equations:

$$\frac{dx_{FC}}{dt} = F_{FC_{prod}} + F_{CEdef_{cyt}} + F_{CEdef_{ER}} - F_{FC_{met}} - F_{CEfor_{cyt}} - F_{CEfor_{ER}}$$

$$\frac{dx_{CE_{cyt}}}{dt} = F_{CEfor_{cyt}} - F_{CEdef_{cyt}} + V_{plasma}\left(F_{CEupt_{hep}} + F_{CEupt_{HDL}}\right)$$

$$\frac{dx_{CE_{ER}}}{dt} = F_{CEfor_{ER}} - F_{CEdef_{ER}} - F_{VLDL-CE}$$

$$\begin{aligned}
\frac{dx_{TG_{cyt}}}{dt} &= F_{TGfor_{cyt}} - F_{TGfor_{ER}} - F_{TGmet_{cyt}} \\
&\qquad + V_{plasma}\left(\frac{F_{FFA_{upt}}}{3} + F_{TGupt_{hep}} + F_{TGhyd_{hep}}\right) \\
\frac{dx_{TG_{ER}}}{dt} &= F_{TGfor_{ER}} - F_{TGfor_{cyt}} - F_{VLDL-TGndnl} \\
\frac{dx_{TGdnl_{cyt}}}{dt} &= F_{TGdnl_{cyt}} - F_{TGdnlmet_{cyt}} + F_{TGdnlfor_{cyt}} - F_{TGdnlfor_{ER}} \\
\frac{dx_{TGdnl_{ER}}}{dt} &= F_{TGdnl_{ER}} + F_{TGdnlfor_{ER}} - F_{TGdnlfor_{cyt}} - F_{VLDL-TGdnl} \\
\frac{dx_{TG_{VLDL}}}{dt} &= \frac{F_{VLDL-TG}}{V_{plasma}} - F_{TGupt_{hep}} - F_{TGupt_{per}} - F_{TGhyd_{hep}} - F_{TGhyd_{per}} \\
\frac{dx_{C_{VLDL}}}{dt} &= \frac{F_{VLDL-CE}}{V_{plasma}} - F_{CEupt_{hep}} - F_{CEupt_{per}} \\
\frac{dx_{C_{HDL}}}{dt} &= F_{CEfor_{HDL}} - F_{CEupt_{HDL}} \\
\frac{dx_{FFA}}{dt} &= F_{FFA_{prod}} - F_{FFA_{upt}}
\end{aligned}$$

Two state variables in the model ($x_{TGdnl_{cyt}}$ and $x_{TGdnl_{ER}}$) represent the fractions of *de novo* produced triglycerides in the cytosol and endoplasmic reticulum, respectively. The blood plasma volume V_{plasma} was assumed to be 1 mL (Rand, 2001).

The HepaLip2 model contains lumped reaction equations, and does for example not describe the dynamics of the amount and activity of the enzymes. The law of mass-action states that the rate of an elementary reaction is proportional to the product of the concentrations of the participating substrates. Consequently, the rate of product formation shows a linear response to increasing substrate. Mathematical models of enzyme-catalyzed reactions often include kinetic equations in which reaction rates saturate and the product formation depends nonlinearly on substrate concentrations. We had tested before if the simplification introduced by mass-action kinetics might impede the accuracy of the model, or if introducing saturable enzyme kinetics (Michaelis-Menten) would make the ADAPT approach obsolete or superfluous. Both hypotheses were rejected, hence a mathematical model based on mass-action kinetics with time varying parameter is sufficient to describe our data and cannot be replaced by Michaelis-Menten kinetics with constant parameters (Tiemann, C. A., 2014).

Table S1. Fluxes included in HepaLip2 model, see **Figure 1** in the main text .

Flux	Name	Equation	Description
f_1	F_{FCprod}	p_1	Hepatic *de novo* synthesis of free cholesterol
f_2	F_{FCmet}	$p_2 \cdot x_{FC}$	Net hepatic catabolism of free cholesterol
f_3	$F_{CEfor_{cyt}}$	$p_3 \cdot x_{FC}$	Hepatic synthesis of cholesteryl ester (cytoplasm)
f_4	$F_{CEdef_{cyt}}$	$p_4 \cdot x_{CE_{cyt}}$	Hepatic conversion of cholesteryl ester (cytoplasm) to free cholesterol
f_5	$F_{CEfor_{ER}}$	$p_5 \cdot x_{FC}$	Hepatic synthesis of cholesteryl ester (ER)
f_6	$F_{CEdef_{ER}}$	$p_6 \cdot x_{CE_{ER}}$	Hepatic conversion of cholesteryl ester (ER) to free cholesterol
f_7	$F_{TGdnl_{cyt}}$	p_7	Hepatic *de novo* synthesis of triglyceride (cytoplasm)
f_8	$F_{TGmet_{cyt}}$	$p_8 \cdot x_{TG_{cyt}}$	Hepatic catabolism of triglyceride (cytoplasm)
f_9	$F_{TGfor_{cyt}}$	$p_9 \cdot x_{TG_{ER}}$	Hepatic transport of triglyceride from the ER to the cytoplasm
f_{10}	$F_{TGdnl_{ER}}$	p_{10}	Hepatic *de novo* synthesis of triglyceride (ER)
f_{11}	$F_{TGfor_{ER}}$	$p_{11} \cdot x_{TG_{cyt}}$	Hepatic transport of triglyceride from the cytoplasm to the ER
f_{12}	$F_{FFA_{upt}}$	$p_{12} \cdot x_{FFA}$	Hepatic uptake of free fatty acid
f_{13}	$F_{FFA_{prod}}$	p_{13}	Net efflux of free fatty acid from peripheral tissues to plasma
f_{14}	$F_{VLDL-TG}$	$p_{14} \cdot (x_{TG_{ER}} + x_{TGdnl_{ER}})$	Hepatic secretion rate of VLDL-triglyceride
f_{15}	$F_{VLDL-CE}$	$p_{15} \cdot x_{CE_{ER}}$	Hepatic secretion rate of VLDL-cholesterol
f_{16}	$F_{TGupt_{hep}}$	$p_{16} \cdot x_{TG_{VLDL}}$	Hepatic uptake of triglyceride via whole-particle uptake
f_{17}	$F_{CEupt_{hep}}$	$p_{16} \cdot x_{C_{VLDL}}$	Hepatic uptake of cholesterol via whole-particle uptake
f_{18}	$F_{TGupt_{per}}$	$p_{17} \cdot x_{TG_{VLDL}}$	Peripheral uptake of triglyceride via whole-particle uptake
f_{19}	$F_{CEupt_{per}}$	$p_{17} \cdot x_{C_{VLDL}}$	Peripheral uptake of cholesterol via whole-particle uptake
f_{20}	$F_{CEfor_{HDL}}$	p_{20}	Peripheral efflux of cholesterol to HDL particles
f_{21}	$F_{CEupt_{HDL}}$	$p_{21} \cdot x_{C_{HDL}}$	Hepatic uptake of HDL-cholesterol
f_{22}	$F_{TGhyd_{hep}}$	$p_{18} \cdot x_{TG_{VLDL}}$	Hepatic uptake of triglyceride via lipolytic enzymes
f_{23}	$F_{TGhyd_{per}}$	$p_{19} \cdot x_{TG_{VLDL}}$	Peripheral uptake of triglyceride via lipolytic enzymes
f_{24}	$F_{apoB_{prod}}$	p_{22}	Hepatic secretion rate of apolipoprotein B
f_{25}	$F_{TGdnlmet_{cyt}}$	$p_8 \cdot x_{TGdnl_{cyt}}$	Hepatic catabolism of *de novo* triglyceride (cytoplasm)
f_{26}	$F_{TGdnlfor_{cyt}}$	$p_9 \cdot x_{TGdnl_{ER}}$	Hepatic transport of *de novo* triglyceride from the ER to the cytoplasm
f_{27}	$F_{TGdnlfor_{ER}}$	$p_{11} \cdot x_{TGdnl_{cyt}}$	Hepatic transport of *de novo* triglyceride from the cytoplasm to the ER
f_{28}	$F_{VLDL-TGndnl}$	$p_{14} \cdot x_{TG_{ER}}$	Hepatic secretion rate of non *de novo* VLDL-triglyceride
f_{29}	$F_{VLDL-TGdnl}$	$p_{14} \cdot x_{TGdnl_{ER}}$	Hepatic secretion rate of *de novo* VLDL-triglyceride

Note $f_{14} = f_{28} + f_{29}$.

2.3 Relating experimental data to model components

An overview of the quantities that were experimentally measured and their relation to corresponding model components is presented in **Table 2** in the main text. A model output y_i was coupled to experimental data $d_{m,i}$ (**Figure 2** in the main text) for the estimation of parameter trajectories.

2.3.1 Calculation of the VLDL particle diameter

The size and composition of VLDL particles change over time. The following approach was used to calculate nascent VLDL particle diameters (D_{VLDL}) in the mathematical model. As each VLDL particle contains one apolipoprotein B particle, the number of triglyceride and cholesterylester molecules per VLDL particle can be determined by correcting the specific lipid fluxes for the number of apolipoprotein B proteins. The core volume of a VLDL particle was subsequently determined assuming a molecular volume of 946.84 mL/mol for triglyceride (TG_{mv}) and a molecular volume of 685.48 ml/mol for cholesterylester (CE_{mv}) (Teerlink et al., 2004). A core radius (R_c) was calculated from the core volume assuming a spherical shape of the VLDL particles. Furthermore, the thickness of the particle membrane (R_s) accounts for an additional two nanometers (Miller and Smith, 1973).

$$D_{VLDL}(t) = 2\left(R_c(t) + R_s\right) \tag{S1a}$$

$$R_c(t) = \sqrt[3]{\frac{3V_c(t)}{4\pi}} \tag{S1b}$$

$$V_c(t) = 10^{21}\frac{TG_{cnt}(t)\cdot TG_{mv} + CE_{cnt}(t)\cdot CE_{mv}}{N_A} \tag{S1c}$$

$$TG_{cnt}(t) = \frac{f_{14}(t)}{f_{24}(t)} \tag{S1d}$$

$$CE_{cnt}(t) = \frac{f_{15}(t)}{f_{24}(t)} \tag{S1e}$$

where N_A is the constant of Avogadro.

2.3.2 Calculation of de novo lipogenesis

Model output y_{11} is the normalized VLDL catabolic rate (CR_{VLDL}), which represents the whole-body capacity to clear lipoproteins from the circulation. The equation is given by:

$$CR_{VLDL}(t) = \frac{p_{16}(t) + p_{17}(t)}{p_{16}(0) + p_{17}(0)} \tag{S2}$$

Model output y_{13} is the fractional contribution of *de novo* lipogenesis (FC_{DNL}). This quantity represents the fraction of the total hepatic triglyceride pool that is obtained via *de novo* lipogenesis. The equation is given by:

$$FC_{DNL}(t) = \frac{x_{TGdnl_{cyt}}(t) + x_{TGdnl_{ER}}(t)}{x_{TG_{cyt}}(t) + x_{TG_{ER}}(t) + x_{TGdnl_{cyt}}(t) + x_{TGdnl_{ER}}(t)} \tag{S3}$$

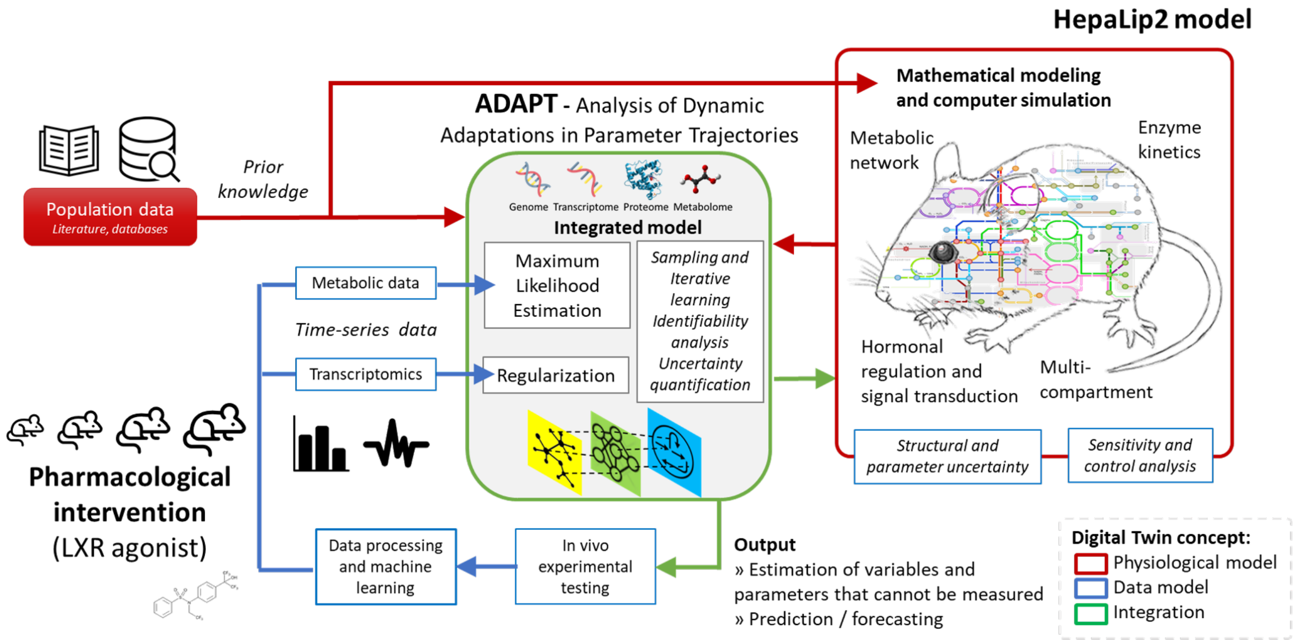

Figure S1. ADAPT methodology workflow. ADAPT combines mechanistic simulation models with machine learning to estimate unobserved system state variables and parameters. Metabolic time-series data is assimilated using Maximum Likehood estimation. Transcriptomics time-series data assimilated via regularization to constrain parameter trajectories.

3 ADAPT

ADAPT combines mechanistic simulation models with machine learning to estimate unobserved system state variables and parameters (**Figure S1**). The computational model functions as a state-estimator and is applied to monitor the effect of therapeutic interventions and detect critical transitions of the system. ADAPT and other data assimilation approaches enable the development of so-called digit twins in which computer simulation models are connected to their biological counterparts by different types of data (van Riel et al., 2020).

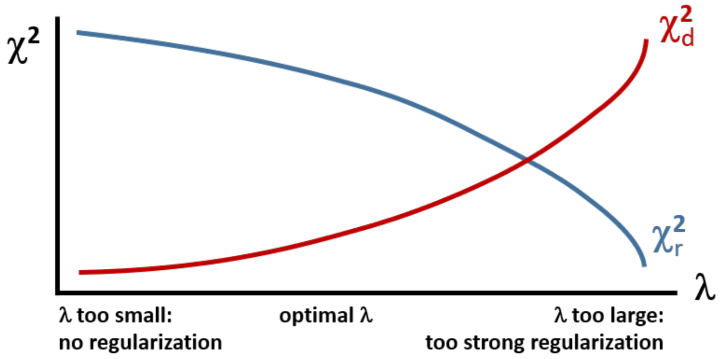

Figure S2. Determination of regularization weight coefficient λ. Trade-off between fitting the data as closely as possible (red line; left side of the horizontal axis) and enforcing smooth parameter trajectories (blue line; right side of the horizontal axis).

3.1 Monte Carlo sampling of gene regularization constants

ADAPT estimates parameter trajectories by minimizing an objective function χ^2 that is composed of the (weighted) sum of squared errors (SSE) of the metabolic data and model outputs (χ^2_d) and a regularization function (χ^2_r) (**Figure S2**). In multi-objective optimization and regularized regression approaches the weights of the different components in the objective function are important meta-parameters of the algorithm that are problem dependent and need to be tuned for adequate performance (Tiemann et al., 2011; Dolejsch et al., 2019).

To assimilate the transcriptomics time-series data, the regularization as originally introduced in ADAPT (Tiemann et al., 2011) has been extended with a second regularization function. Two hyper-parameters are introduced: λ_{g_1} and λ_{g_2} (Equation 3 in the main text). The influence of the regularization constants λ_{g_1} and λ_{g_2} on the estimation of the parameter trajectories was investigated using a Monte Carlo approach. Combinations of random values for λ_{g_1} and λ_{g_2} were sampled from a log-uniform distribution (10^{-12} to 10^{-2}). Subsequently, ADAPT was performed for each sampled combination. Note that the initial values for the parameters and the cubic smoothing splines were selected randomly as well. Parameter trajectories were estimated using $N_t = 200$ time intervals. Finally, a collection of $20,000$ parameter trajectory sets was obtained. The effect of the regularization constants λ_{g_1} and λ_{g_2} on the objective function was investigated by summation of the three components of the objective function for the entire treatment period:

$$\Upsilon_d(\hat{\vec{p}}) = \sum_{n=1}^{N_t} \chi^2_d(\hat{\vec{p}}(n\Delta t)) \tag{S4}$$

$$\Upsilon_{g1}(\hat{\vec{p}}) = \sum_{n=1}^{N_t} \chi^2_{g1}(\hat{\vec{p}}(n\Delta t)) \tag{S5}$$

$$\Upsilon_{g2}(\hat{\vec{p}}) = \sum_{n=1}^{N_t} \chi^2_{g2}(\hat{\vec{p}}(n\Delta t)) \tag{S6}$$

where χ^2_d is the (weighted) sum of squared errors of metabolic data and model outputs (Equation 7 in the main text), $\chi^2_{g_1}$ reflects the temporal correlation between parameter trajectories and gene expression profiles (Equation 8 in the main text), and $\chi^2_{g_2}$ penalizes parameter fluctuations (Equation 11 in the main text).

Figure S3 presents 2D histograms of the sampled regularization constants, where the intensity indicates corresponding mean values of Υ_d (**Figure S3A**), Υ_{g_1} (**Figure S3B**) and Υ_{g_2} (**Figure S3C**). Note the intensities in the three figure panels have a different (log_{10}) scale. Several observations can be made from these graphs. First, the variation in the data error Υ_d is relatively small within the selected range of regularization constants. All solutions describe the experimental data adequately (simulations are within the 95% confidence intervals of the data). However, when λ_{g_2} is increased towards and beyond 10^{-2}, the data error becomes considerably higher. Only a negligible amount of acceptable solutions were found for λ_{g_1} and / or λ_{g_2} larger than 10^{-2}. Secondly, the light-gray region in the bottom-right part of **Figure S3B** clearly illustrates for which combinations of regularization constants $\chi^2_{g_1}$ becomes effective and parameter-gene couples start to display temporal correlation. Finally, **Figure S3C** illustrates that a small value for λ_{g_1} is sufficient to reduce unnecessary parameter trajectory fluctuations.

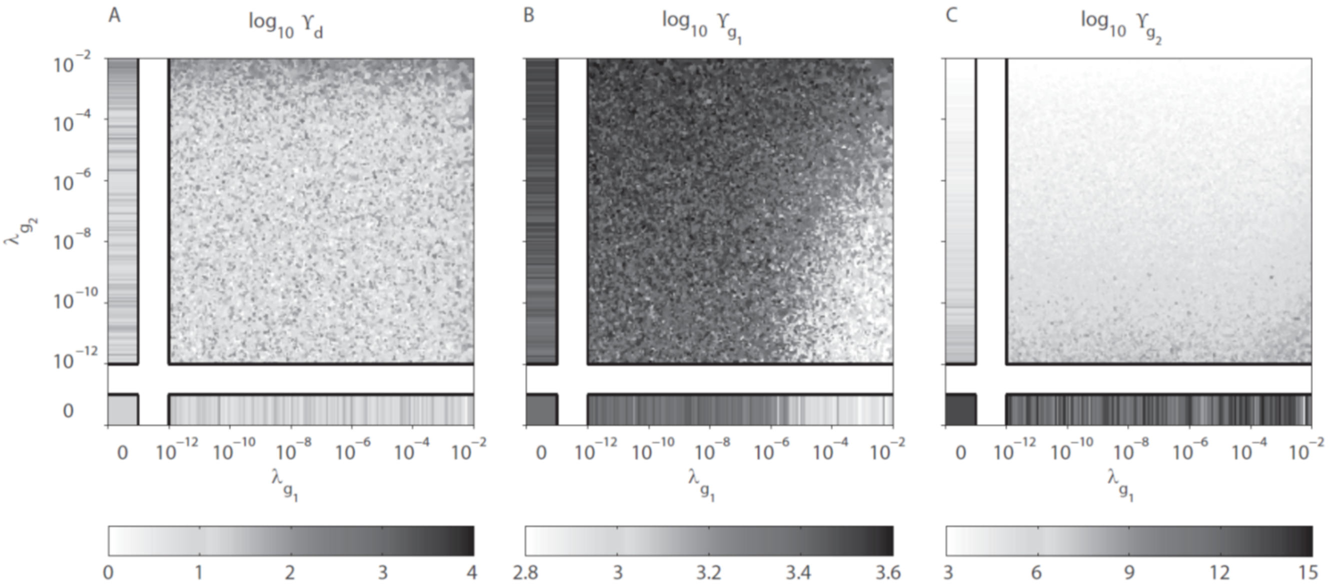

Figure S3. Monte Carlo sampling of gene regularization constants. The influence of the regularization constants λ_{g1} and λ_{g2} (in Equation 3 in the main text) on the estimation of the parameter trajectories was investigated using a Monte Carlo approach. ADAPT was performed for $20,000$ random combinations for λ_{g1} and λ_{g2} (sampled from a log-uniform distribution between 10^{-12} and 10^{-2}). The figures represent 2D histograms of the sampled regularization constants, where the intensity indicates corresponding mean values of Υ_d (A), Υ_{g1} (B) and Υ_{g2} (C), defined in equations (S4)-(S6). The higher Υ is, the larger the contribution of that component is in the objective function χ^2 summed over the complete time course. Results for $\lambda_{g1} = 0$ (no penalty if parameter trajectories and temporal gene expression do not correlate) or $\lambda_{g2} = 0$ (no penalty on parameter changes) are shown to the left and at the bottom of each panel, respectively.

A limitation of using one regularization constant for all included genes, is that a single parameter-gene couple could seriously restrict the value for this constant. In principle, the current approach can be extended by introducing a regularization constant for each parameter-gene couple.

3.2 Classification of trajectory solution groups

The characteristics of trajectory solutions corresponding to a specific combination of gene regularization constants were investigated. Of particular interest are the solutions with a low value of Υ_{g_1} (**Figure S3B**, bottom-right part). Parameter trajectories corresponding to these solutions display a high temporal correlation with the included gene expression data. In subsequent analyses we compared different groups of trajectory solutions corresponding to different values of Υ_{g_1}. We use the following notation: a group of trajectory solutions is denoted by G_i where i represents the fraction of all $20,000$ solutions with lowest Υ_{g_1} values. A lower value of i indicates a group with lower values for Υ_{g_1} (higher temporal correlations) and hence a more effective integration of the gene expression data. Furthermore, G^0 is defined as the group of solutions corresponding to $\lambda_{g_1} = \lambda_{g_2} = 0$ (solutions obtained without integration of gene expression data and without penalty on parameter fluctuations).

As an example, **Figure S4** shows the temporal correlation between parameter-gene couples for the full treatment period for group $G_{0.05}$ (**Figure S4**, top part) and group G^0 (**Figure S4**, bottom part). In some cases parameter-gene couples in G^0 already displayed a (high) temporal correlation when no gene expression data was included (for instance $c_{8,1\cdots5}$ and $c_{9,1\cdots5}$). As expected, in many cases a large increase in temporal correlation between the assigned parameter-gene couples was obtained when gene expression data was included ($G_{0.05}$). Interestingly, in one case (couple c_5) a predominantly negative correlation was observed for all solution groups. The gene expression data imposes soft constraints on the parameter trajectories. Hence, parameter trajectories and corresponding gene expression levels do not necessarily have to display temporal correlation when this is in contradiction to the metabolic data. In this specific case, the observed negative correlation of couple c_5 can be explained as follows. Couple c_5 concerns parameter p_{22} (or flux f_{24}) which represents the VLDL particle secretion (or apolipoprotein B secretion) to the plasma. In **Figure S7** (and Tiemann et al. (2013)) we show that the VLDL particle secretion decreased rapidly over one week of treatment and subsequently stabilized upon prolonged treatment. The predicted adaptations of this flux were constrained by experimental data of the VLDL particle size (y_9) and the VLDL-TG production flux (y_{10}). In contrast to the apolipoprotein B secretion flux, the expression of the corresponding gene in the liver was not reduced (**Figure 3** in the main text). This is not surprising as apolipoprotein B expression is known to be regulated post-transcriptionally (Adeli et al., 1995; Dixon and Ginsberg, 1993; Pullinger et al., 1989).

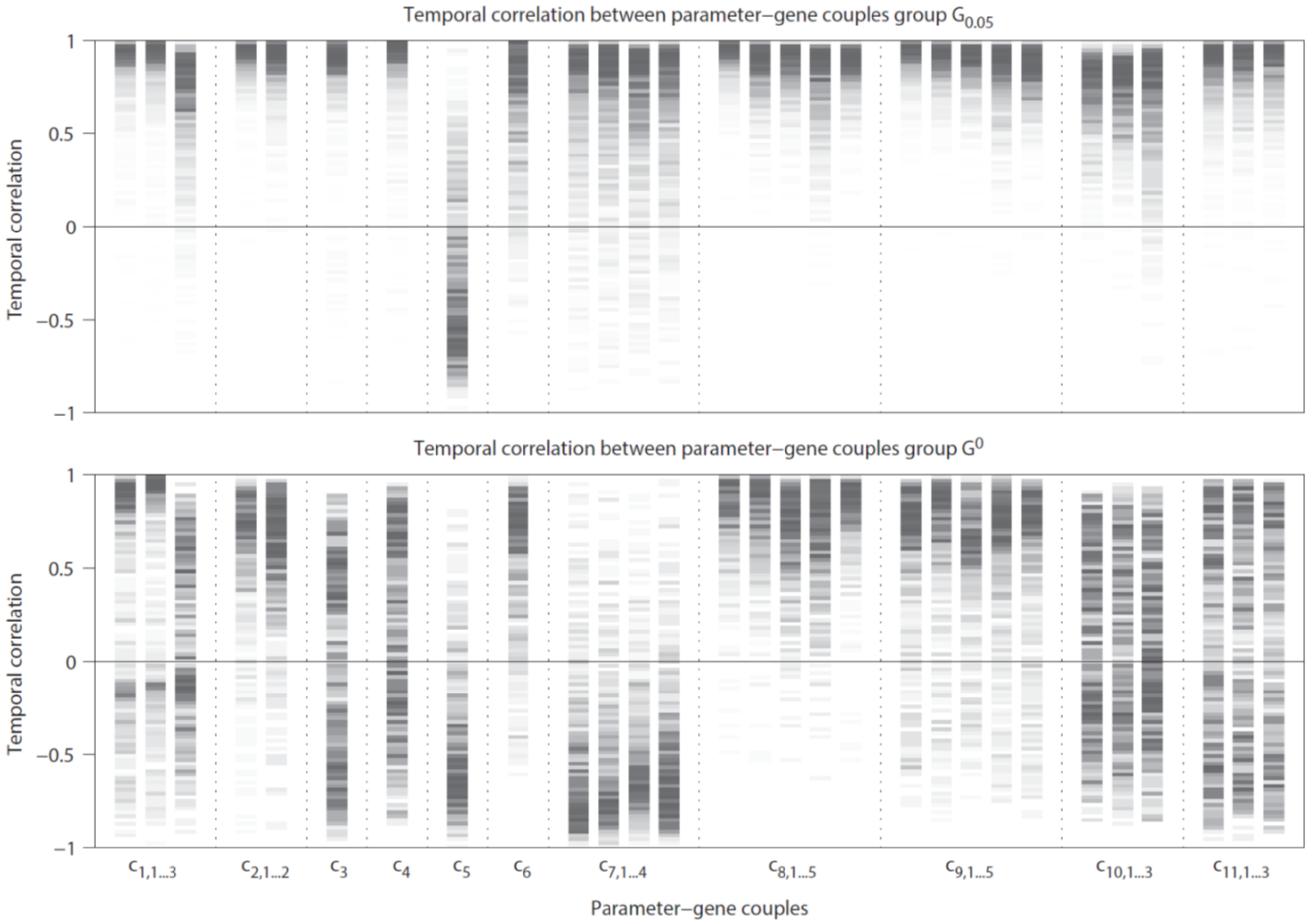

Figure S4. Temporal correlation between parameters and gene expression data. Histograms of Pearson correlation coefficients between parameter-gene couples for the full treatment period for group $G_{0.05}$ (top) and group G^0 (bottom). $G_{0.05}$ consists of 5% $(1,000)$ of all $20,000$ trajectory solutions with the lowest Υ_{g_1} values, hence largest correlation between parameter trajectories and temporal gene expression. G^0 is the group of solutions for $\lambda_{g_1} = \lambda_{g_2} = 0$ (without integration of gene expression data and without penalty on parameter fluctuations). A darker color represents a higher density of solutions with that specific Pearson correlation coefficient. The parameter-gene couples are presented in **Table 3** in the main text.

3.3 Integration of gene data constrains metabolic predictions

The reduction in the uncertainty in model predictions by implicit integration of gene expression data was investigated. To assess whether the variance of a specific model prediction C at time step n is reduced in group G_i compared to group G_j, the following measure for variance reduction V^r was defined:

$$V^r_{ij}(n\Delta t) = 1 - \frac{\text{Var}(\vec{C}_{G_i}(n\Delta t))}{\text{Var}(\vec{C}_{G_j}(n\Delta t))} \tag{S7}$$

where Var represents the variance operator, and $\vec{C}_{G_i}(n\Delta t)$ the vector of predictions of output C at time step n from group G_i. Note that the maximal value for $V^r_{ij}(n\Delta t)$ is 1 (maximal attainable variance reduction), and $V^r_{ij}(n\Delta t) = 0$ indicates that no variance reduction is obtained. The variance reduction in model predictions was calculated for different groups G_i compared to G^0, with $0.05 \leq i \leq 1$. In many cases a

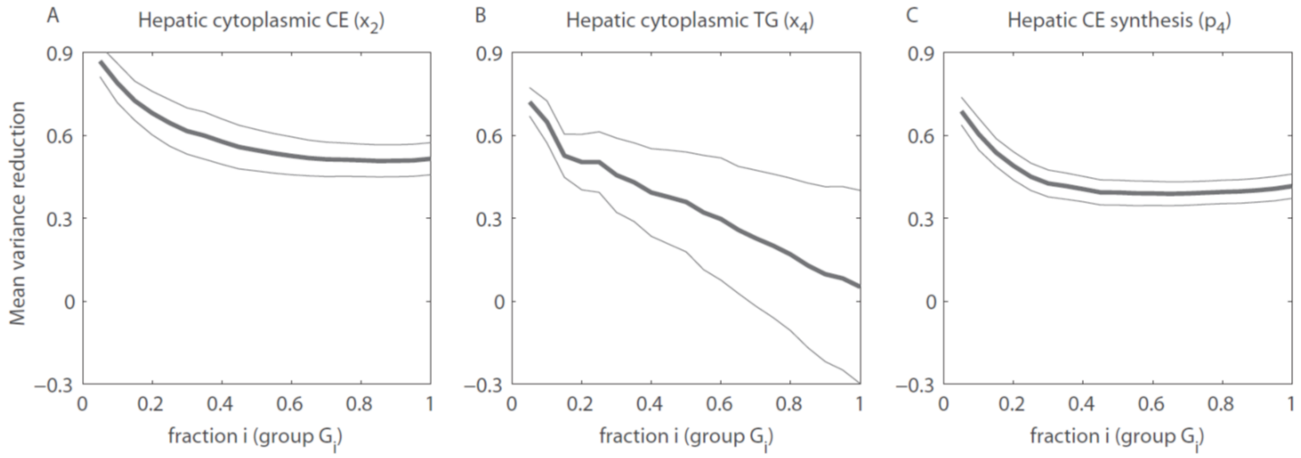

Figure S5. Correlation of parameter trajectories and temporal gene expression reduces model variance. The variance reduction (according to equation S7) in model estimates was calculated for different solution groups G_i (with $0.05 \leq i \leq 1$). For many model estimates a reduction in variance was obtained by regularization using gene expression data. Typically, the reduction in variance increased with decreasing values for i. High correlation between parameter trajectories and temporal gene expression means the penalty introduced by χ^2_{g1} is small. Those solutions are found in the groups G_i with the smaller values for i. Three examples are depicted: hepatic cytosolic cholesterylester (A), hepatic cytosolic triglyceride (B), and hepatic cholesterylester synthesis (C). The thick lines indicate the mean, whereas the thin lines represent the standard deviation.

reduction in prediction variance was obtained. Furthermore, in general a higher variance reduction was obtained for lower values of i. **Figure S5** shows three examples: hepatic cytosolic cholesterylester, hepatic cytosolic triglyceride, and hepatic cholesterylester synthesis. Model estimates of group $G_{0.05}$ are presented in the main text in more detail.

3.4 Numerical aspects

Parameter updates are preferred such that resulting parameter trajectories and corresponding gene expression profiles display temporal correlation. This was effectuated by maximizing the Pearson correlation coefficient. Other correlation metrics can be incorporated as well. However, some correlation metrics are not usable in combination with certain numerical optimization algorithms. Many optimization algorithms calculate the second-order derivative of the objective function with respect to the parameters (or an approximation thereof) to propose a new optimization step (Fletcher, 2013; Snyman, 2005). Hence, for these methods the correlation metric must be second-order differentiable with respect to the parameters. For some metrics this is not possible, such as the Spearman correlation coefficient and the Kendall tau correlation coefficient, as their calculation involves the ranking of variables (Kendall, 1948). For these cases one should resort to non-gradient based optimization methods, *e.g.*, simplex optimization methods such as Nelder-Mead (Nelder and Mead, 1965).

Another numerical aspect to consider is the use of a variable step integration method to solve the ODE model for each of the time segments Δt (**Figure S6**). We used SUNDIALS CVode and built in Matlab solvers like ode15s. If Δt is chosen sufficiently small, the ODE's in principle could also be solved using a fixed step integration algorithm, like forward Euler. In such case integration of the ODE's could be combined with updating of the model parameters in a single numerical algorithm.

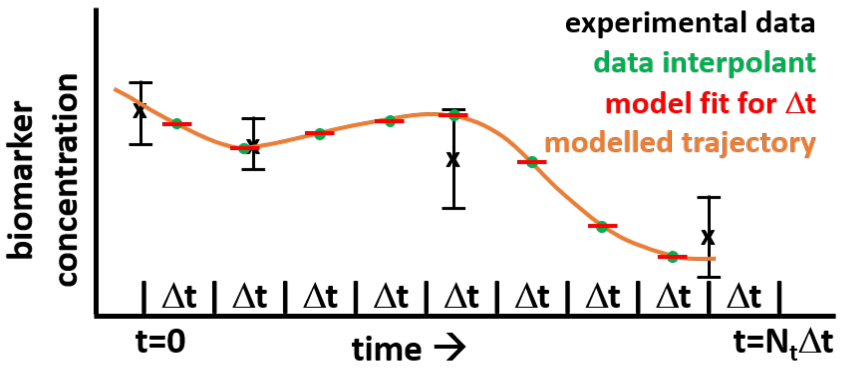

Figure S6. ADAPT simulation. Discretization of the time span. The original experimental data are depicted by black error bars. For a selected data interpolant (see **Figure 9B** in the main text) the values at discrete, equidistant time points $n\Delta t$, $n = 0, .., N_t$ are selected (green dots). The model (red) is fitted to these data interpolants per time segment. For each time segment, the model parameters are re-estimated taking the parameter values of the previous time segment as initial guesses, hence each time segment is modelled using a specific parameter set (red). For each time segment the ODE's are solved given that specific parameter set, using a variable step integration method and the piece-wise model solutions become a fully continuous, smooth trajectory (orange).

4 HEPATIC TRIGLYCERIDE ACCUMULATION

ADAPT simulation of HepaLip2 provides estimates for system variables that were not experimentally observed, such as the synthesis rate and composition of VLDL particles (**Figure S7**). As observed before (Tiemann et al., 2013), VLDL particle secretion is reduced upon LXR activation. Although the secretion of VLDL particles decreased, an increased release of VLDL-TG to the plasma was experimentally observed (**Figure S7B**). Similarly, the computational analysis showed an increased production of VLDL-CE to the plasma (**Figure S7C**). According to the model the progressive increase of these fluxes was facilitated by an increased loading of triglycerides and cholesterol onto VLDL particles (**Figure S7D,E**). These predictions were obtained using only the metabolic data as input for ADAPT.

Gene expression data was integrated in ADAPT to further constrain metabolic predictions. It was estimated that hepatic triglycerides are mainly stored in the cytosol, as reported in the main text (Section 2.8). The parameter and flux trajectories were investigated to determine which processes are responsible for the observed compartmentalization of hepatic triglycerides between cytosolic and nascent VLDL fractions. It appeared that the calculation of constrained predictions for the nascent VLDL triglyceride content was enabled by two factors. First, the nascent VLDL triglyceride content is co-determined by the hepatic capacity to load these triglycerides onto nascent produced VLDL particles (p_{14}). The loading capacity depends among other things on the activity level of the microsomal transfer protein (Mtp) (Aggerbeck et al., 1992; Hussain et al., 2012). The expression level of the *Mtp* gene was experimentally measured (**Figure 3** in the main text) and coupled to parameter p_{14} (couple $c_{3,1}$, **Table 3** in the main text). A second factor is the VLDL-TG production flux which increases progressively during the treatment (**Figure S7**). The VLDL-TG production flux is (mathematically) related to the aforementioned loading capacity and the nascent VLDL triglyceride content. **Figure S8** (left) shows a scatter plot of all $20,000$ solutions at $t = 21$ days of the normalized loading capacity (p_{14}) and the nascent VLDL triglyceride content ($x_5 + x_7$). The color indicates the temporal correlation of $c_{3,1}$. As the *Mtp* gene expression level increased during the treatment, solutions with an increased lipid loading capacity displayed a high temporal correlation. An increased lipid loading

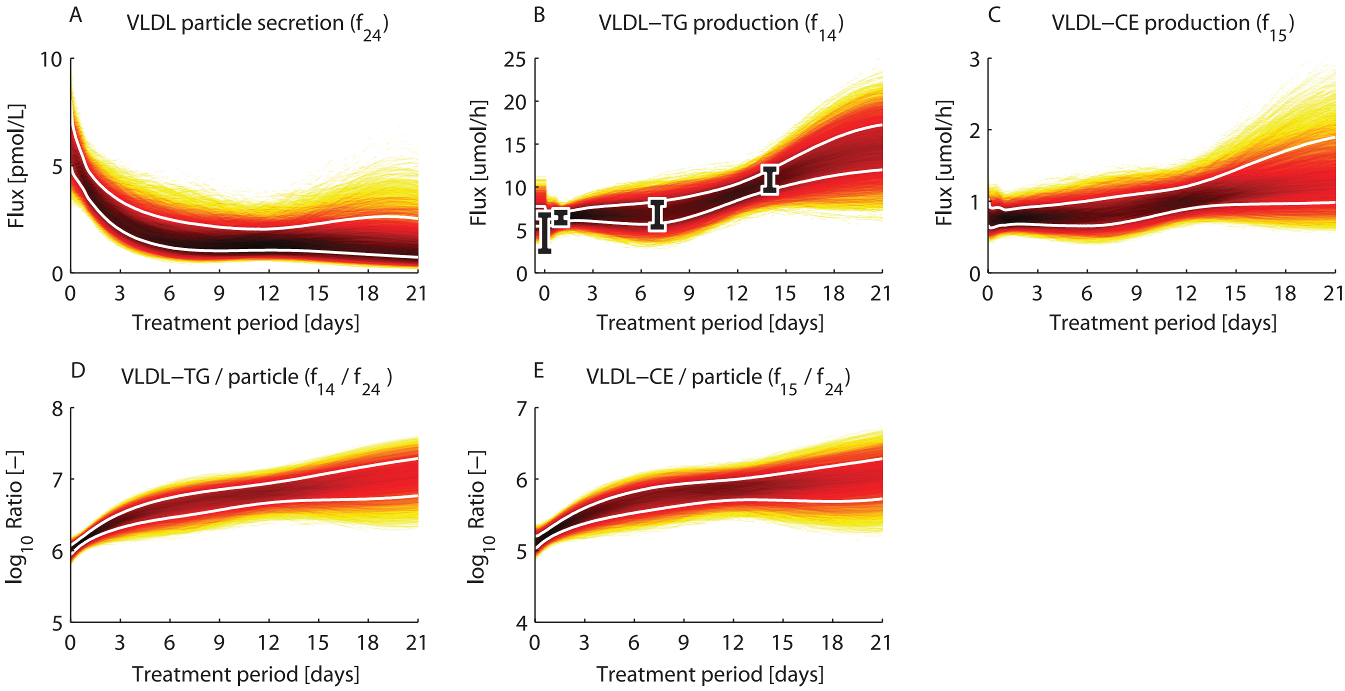

Figure S7. The VLDL flux and composition upon LXR activation. 2D histograms were calculated from the $10,000$ acceptable sets to determine the density of trajectories during the treatment period. A darker color represents a higher density of trajectories in that specific region and time point. The white lines enclose the central 67% of the densities. A) VLDL particle secretion. B) VLDL-TG production. The data is represented by mean $\pm$ standard deviation. C) VLDL-CE production. D) Ratio of VLDL-TG production to VLDL particle secretion. E) Ratio of VLDL-CE production to VLDL particle secretion. These results were obtained by using the metabolic data (**Figure 2** in the main text) as input for ADAPT; gene expression data was not included.

capacity is in turn associated with low nascent VLDL triglyceride levels (approximately at the level of untreated controls). Consequently, the increased triglyceride fluxes should be stored in the cytosol, to obey these mathematical relations. **Figure S8** (right) shows the transition of the temporal correlation $c_{3,1}$ as function of the nascent VLDL triglyceride content during the treatment period. Well-defined ranges of the nascent VLDL triglyceride content can be observed for each stage during the treatment that coincide with high temporal correlations of couple $c_{3,1}$.

To study the origin of LXR induced hepatic steatosis the sum of all fluxes contributing to the hepatic triglyceride pool F_a and the sum of fluxes that catabolize hepatic triglycerides F_s were calculated as follows:

$$F_a(t) = F_{TGdnl_{cyt}}(t) + F_{TGdnl_{ER}}(t) + V_{plasma}\left(\frac{F_{FFA_{upt}}(t)}{3} + F_{TGupt_{hep}}(t) + F_{TGhyd_{hep}}(t)\right) \quad \text{(S8)}$$

$$F_s(t) = F_{VLDL-TG}(t) + F_{TGmet_{cyt}}(t) + F_{TGdnlmet_{cyt}}(t) \quad \text{(S9)}$$

Results are reported in the main text (**Figure 7**).

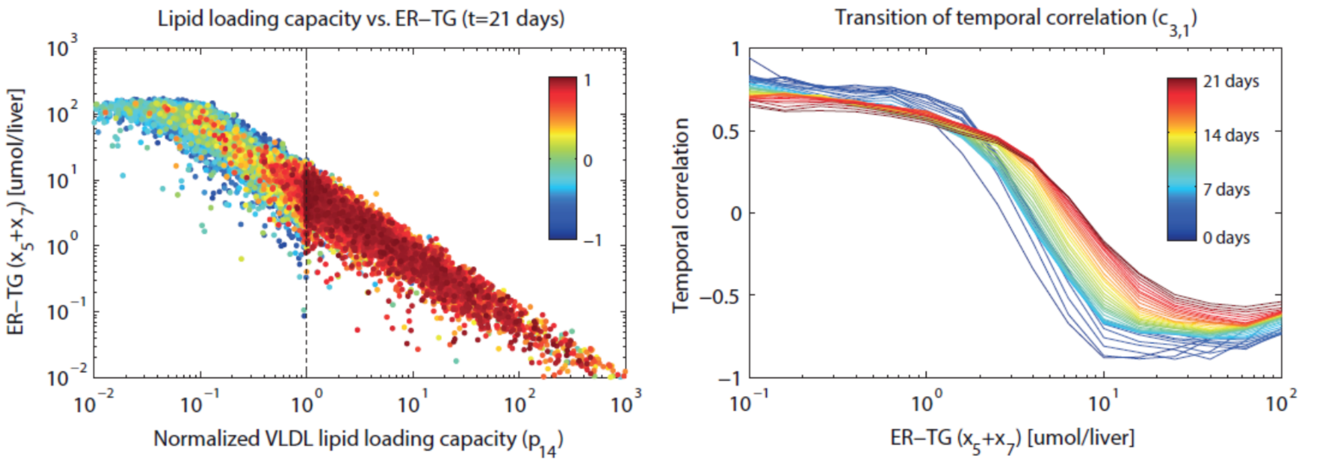

Figure S8. The hepatic lipid loading capacity to lipoproteins is increased. Left) Scatter plot of all $20,000$ solutions at $t = 21$ days of the normalized loading capacity of lipid onto VLDL particles (p_{14}) and the nascent VLDL triglyceride content (ER-TG, $x_5 + x_7$). The color indicates the temporal correlation of couple $c_{3,1}$, which is parameter p_{14} with gene *Mtp*. Solutions with an increased lipid loading capacity (to the right of the vertical dashed line) display a high temporal correlation with expression of *Mtp*, which also increased during treatment. Right) Transition of the temporal correlation of $c_{3,1}$ as function of the nascent VLDL triglyceride content (ER-TG) during the treatment period. The color of the lines indicates a specific time point during the treatment. Throughout the complete time course solutions with the highest temporal correlation correspond to low nascent VLDL TG levels.

REFERENCES

Adeli, K., Mohammadi, A., and Macri, J. (1995). Regulation of apolipoprotein b biogenesis in human hepatocytes: posttranscriptional control mechanisms that determine the hepatic production of apolipoprotein b-containing lipoproteins. *Clinical biochemistry* 28, 123–130

Aggerbeck, L., Bouma, M., Eisenberg, C., Munck, A., Hermier, M., Schmitz, J., et al. (1992). Absence of microsomal triglyceride transfer protein in individuals with abetalipoproteinemia. *Science* 258, 999–1001

Chao, F.-F., Stiers, D. L., and Ontko, J. A. (1986). Hepatocellular triglyceride synthesis and transfer to lipid droplets and nascent very low density lipoproteins. *Journal of Lipid Research* 27, 1174–1181

Dixon, J. L. and Ginsberg, H. (1993). Regulation of hepatic secretion of apolipoprotein b-containing lipoproteins: information obtained from cultured liver cells. *Journal of lipid research* 34, 167–179

Dolejsch, P., Hass, H., and Timmer, J. (2019). Extensions of l1 regularization increase detection specificity for cell-type specific parameters in dynamic models. *BMC bioinformatics* 20, 395. doi:10.1186/s12859-019-2976-1

Fletcher, R. (2013). *Practical methods of optimization* (Chichester: John Wiley & Sons)

Gibbons, G., Islam, K., and Pease, R. (2000). Mobilisation of triacylglycerol stores. *Biochim Biophys Acta* 1483, 37–57

Hussain, M. M., Rava, P., Walsh, M., Rana, M., Iqbal, J., et al. (2012). Multiple functions of microsomal triglyceride transfer protein. *Nutr Metab (Lond)* 9, 14

Kendall, M. G. (1948). *Rank correlation methods.* (New York: Griffin)

Miller, A. and Smith, L. (1973). Activation of lipoprotein lipase by apolipoprotein glutamic acid. *J Biol Chem* 248, 3359–3362

Nelder, J. A. and Mead, R. (1965). A simplex method for function minimization. *The computer journal* 7, 308–313

Pullinger, C. R., North, J. D., Teng, B.-B., Rifici, V. A., De Brito, A. R., and Scott, J. (1989). The apolipoprotein b gene is constitutively expressed in hepg2 cells: regulation of secretion by oleic acid, albumin, and insulin, and measurement of the mrna half-life. *Journal of lipid research* 30, 1065–1077

Rand, M. (2001). Handling, restraint, and techniques of laboratory rodents. *Department of Animal Care, University of Arizona*

Snyman, J. A. (2005). *Practical mathematical optimization: an introduction to basic optimization theory and classical and new gradient-based algorithms*, vol. 97 (New York: Springer)

Teerlink, T., Scheffer, P., Bakker, S., and Heine, R. (2004). Combined data from LDL composition and size measurement are compatible with a discoid particle shape. *J Lipid Res* 45, 954–966

Tiemann, C., Vanlier, J., Hilbers, P., and van Riel, N. (2011). Parameter adaptations during phenotype transitions in progressive diseases. *BMC Sys Biol* 5, 174

Tiemann, C., Vanlier, J., Oosterveer, M., Groen, A., Hilbers, P., and van Riel, N. (2013). Parameter trajectory analysis to identify treatment effects of pharmacological interventions. *PLoS computational biology* 9, e1003166

Tiemann, C. A. (2014). *Computational analysis of adaptations during disease and intervention*. Ph.D. thesis, Technische Universiteit Eindhoven

van Riel, N. A., Mueller, R., and Dall'Ara, E. (2020). The Digital Mouse: why computational modelling of mouse models of disease can improve translation. *bioRxiv* , 2020.05.04.075812doi:10.1101/2020.05.04.075812. Publisher: Cold Spring Harbor Laboratory Section: New Results
